# Controllable Iterative β-Glucosylation from UDP-Glucose by *Bacillus cereus* Glycosyltransferase GT1: Application for the Synthesis of Disaccharide-Modified Xenobiotics

**DOI:** 10.1021/acs.jafc.1c05788

**Published:** 2021-11-24

**Authors:** Jihye Jung, Doreen Schachtschabel, Michael Speitling, Bernd Nidetzky

**Affiliations:** † Austrian Centre of Industrial Biotechnology, A-8010 Graz, Austria; ‡ Institute of Biotechnology and Biochemical Engineering, NAWI Graz, TU Graz, A-8010 Graz, Austria; § BASF SE, Carl-Bosch-Strasse 38, 67056 Ludwigshafen, Germany

**Keywords:** Leloir glycosyltransferases, sugar nucleotide, iterative glycosylation, disaccharide modification, xenobiotics

## Abstract

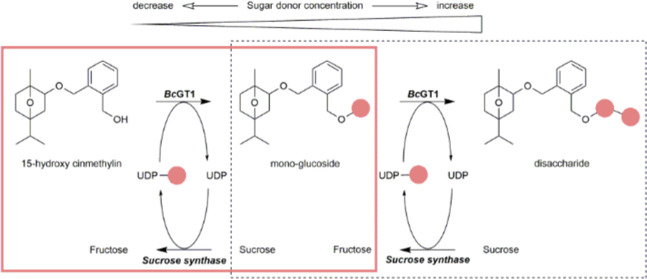

Glycosylation in natural product metabolism and xenobiotic detoxification often leads to disaccharide-modified metabolites. The chemical synthesis of such glycosides typically separates the glycosylation steps in space and time. The option to perform the two-step glycosylation in one pot, and catalyzed by a single permissive enzyme, is interesting for a facile access to disaccharide-modified products. Here, we reveal the glycosyltransferase GT1 from *Bacillus cereus* (*Bc*GT1; gene identifier: KT821092) for iterative *O*-β-glucosylation from uridine 5′-diphosphate (UDP)-glucose to form a β-linked disaccharide of different metabolites, including a C15 hydroxylated detoxification intermediate of the agricultural herbicide cinmethylin (15HCM). We identify thermodynamic and kinetic requirements for the selective formation of the disaccharide compared to the monosaccharide-modified 15HCM. As shown by NMR and high-resolution MS, β-cellobiosyl and β-gentiobiosyl groups are attached to the aglycone’s O15 in a 2:1 ratio. Glucosylation reactions on methylumbelliferone and 4-nitrophenol involve reversible glycosyl transfer from and to UDP as well as UDP-glucose hydrolysis, both catalyzed by *Bc*GT1. Collectively, this study delineates the iterative β-d-glucosylation of aglycones by *Bc*GT1 and demonstrates applicability for the programmable one-pot synthesis of disaccharide-modified 15HCM.

## Introduction

**Figure 1:**
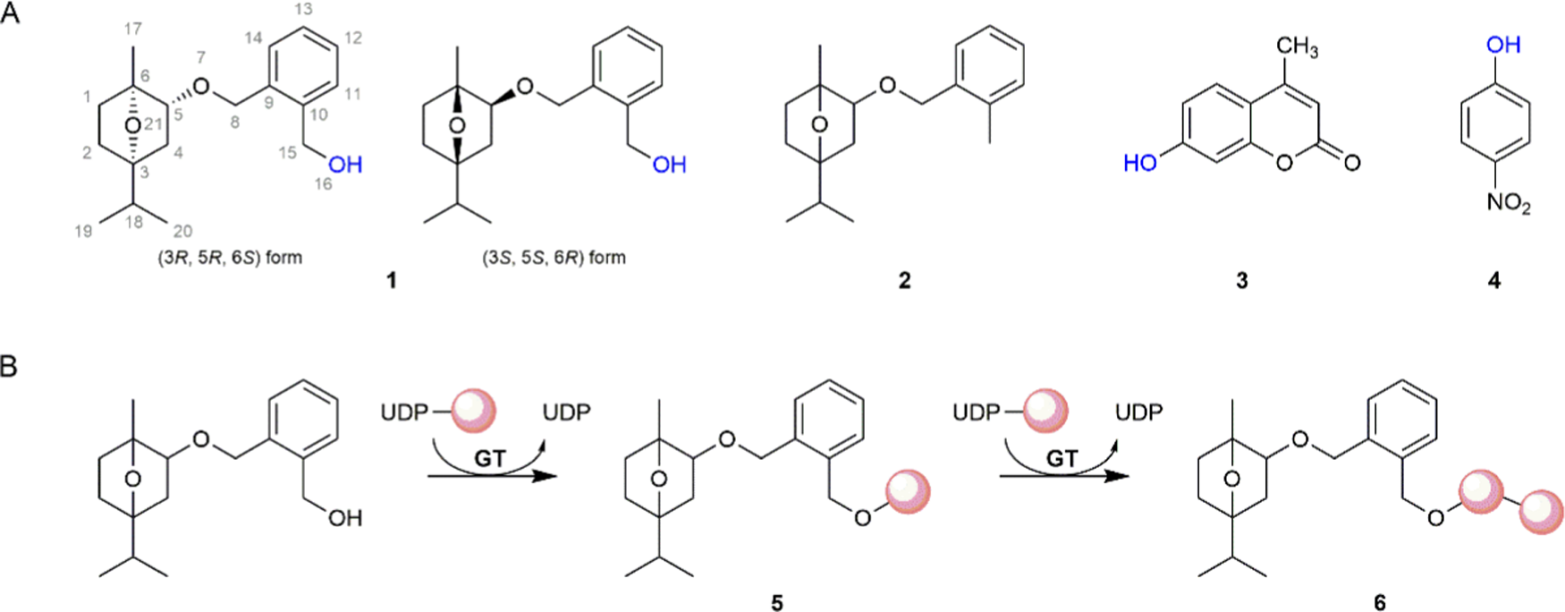
Enzymatic iterative glycosylation of *Bc*GT1 to form disaccharide-modified products. (A) Chemical structures of 15-hydroxy cinmethylin (**1**, 15HCM), cinmethylin (**2**, CM), 4-methylumbelliferone (**3**, 4MU), and 4-nitrophenol (**4**, 4NP). 15HCM, 4MU, and 4NP were used as acceptors of glycosylation by *Bc*GT1 in this study. (B) Iterative glycosylation of 15HCM by *Bc*GT1. *Bc*GT1 catalyzes the transfer of glucosyl residue (red balls) from UDP-glucose to 15HCM, releasing 15HCM-β-d-glucoside (**5**), and to 15HCM-β-d-glucoside, forming 15HCM β-d-glucosyl-β-d-glucoside (**6**, disaccharides).

Glycosylation is a widespread type of chemical conjugation performed on small molecules in natural product biosynthesis^1−3^ and metabolite detoxification.^1,3^ Attachment of sugar residue(s) increases the solubility, often defines the bioactivity, and directs the cellular targeting of the metabolite.^4,5^ In biology, the task of glycosylation is handled by a class of sugar nucleotide-dependent glycosyltransferases.^4,5^ These enzymes use sugar nucleotides as donors for glycosyl transfer to acceptors.^4,5^ Glycosyltransferases offer precise α/β stereocontrol of the glycosylation but differ widely in their substrate scope.^4−6^ For example, detoxifying glycosyltransferases are often highly permissive regarding the acceptor substrates used.^3,4^ Assayed *in vitro*, some glycosyltransferases of secondary metabolism can use a large diversity of donor and acceptor substrates.^3,7^ Due to the interplay of different glycosyltransferases in biosynthesis, the glycosylation on small molecules can give rise to considerable structural diversity.^8,9^ Products can be glycosylated at multiple positions, exhibit a disaccharide, or even an oligosaccharide, attached to the aglycone, or feature both modifications at the same time.^8,9^ Among the natural products, many (e.g., antibiotics like vancomycin;^10,11^ flavonoids like quercetin or luteolin;^12,13^ fragrances and flavors like geraniol^1,5,14^) are found in different glycoside forms and show modulation in function or potency due to change in glycosylation pattern. The steviol glycosides imparting intense sweetness to extracts of the *Stevia* plant are *bis*-glycosides, with a disaccharide (stevioside) or trisaccharide (rebaudioside A) attached to the diterpene aglycone.^15,16^ Our interest here was on metabolite glycosylation with a disaccharide unit, which is chemically challenging to install and not well explored for glycoside synthesis. It prompted a study on glycosyltransferase-catalyzed preparation of disaccharide-modified xenobiotics, with the purpose of establishing a facile and broadly applicable route for metabolite neo-glycosylation.^10,17^ Besides being widespread in natural products, glycosylation with simple β-d-glucose disaccharides (e.g., β-gentiobiosyl, β-d-glucosyl-(1→6)-β-d-glucosyl) plays a significant role in the detoxification of herbicides (e.g., Picloram;^18^ Diphenamid;^19^ 3-phenoxy benzoic acid^20,21^ derived from pyrethroids) in plants. The major target of our inquiry was 15-hydroxy cinmethylin (15HCM; **1**, Figure 1), which is phase I detoxification metabolite of the pre-emergence herbicide cinmethylin (CM; **2**, Figure 1).^22−24^ CM is a benzyl ether derivative of the natural terpene 1,4-cineole that is currently used in a commercialized product (Luximax) for integrated weed and grass management.^22,25^ Besides their importance as analytical reference, disaccharide glucosides of 15HCM have interest for the evaluation of biological efficacy and for the analysis of environmental safety related to CM metabolism.

The synthetic assembly of disaccharide glycosides usually requires a sequential glycosylation procedure with temporal coordination, and potentially spatial separation, of the individual glycosylation steps used.^13,26^ Specific glycosyltransferases might be used in a one-pot linear cascade reaction in which the 15HCM is first glycosylated and the disaccharide then formed on the incipient 15HCM glucoside (**5**, Figure 1). However, such two-enzyme cascade transformations usually involve a large parameter space for optimization and are thus challenging to control for synthetic efficiency.^27,28^ The option to perform the iterative glycosylation by a single permissive enzyme is important for facile access to disaccharide-modified metabolites.

In this study, we identified the glycosyltransferase GT1 from *Bacillus cereus* (*Bc*GT1; GenBank identifier: KT821092) for iterative *O*-β-glucosylation from uridine 5′-diphosphate (UDP)-glucose to build β-linked disaccharide on different metabolites (15HCM, **1**; 4-methylumbelliferone, 4MU, **3**; 4-nitro-phenol, 4NP, **4**; Figure 1). The enzyme was originally identified for glycosylation of flavonoids (e.g., kaempferol, quercetin, apigenin, genistein, naringenin, luteolin).^29^*Bc*GT1 was promising for the current research by virtue of its permissive nature, previously assessed for the formation of mono- and di-glucosylated products of various *O*- and *S*-acceptor substrates (e.g., fluorescein methyl ester, 17-β-estradiol, honokiol, magnolol, *p*-nitrophenol, magnolol, 7-mercapto-4-methylcoumarin, *p*-nitrothiophenol, *p*-thiocresol).^30^ Screening of glycosyltransferases for β-d-mono-glucosylation of 15HCM from UDP-glucose has recently shown the disaccharide-modified product formed in low yield (∼11%) from the *Bc*GT1 reaction.^31^ Based on the comprehensive analysis of time courses for the enzymatic glycosylation of 15HCM combined with detailed product characterization with high-resolution NMR and MS, we here reveal thermodynamic and kinetic requirements for the selective formation of the disaccharide-modified 15HCM in near-quantitative yield. Collectively, our study shows the iterative β-d-glycosylation of aglycones by *Bc*GT1 and demonstrates synthetic applicability of the enzymatic reaction for programmable one-pot disaccharide modification of 15HCM.

## Materials and Methods

### Chemicals and Reagents

Chemicals were from Carl Roth (Karlsruhe, Germany) and Sigma-Aldrich (Vienna, Austria). 4MU (**3**, Figure 1; ≥98%), 4NP (**4**, Figure 1; ≥99%) and 4NP-β-d-glucoside (≥98%) were from Sigma-Aldrich. 4MU-β-d-glucoside, UDP disodium salt, and UDP-glucose disodium salt were from Carbosynth (Compton, U.K.). 15HCM (**1**, Figure 1; ≥99.5%) and 15HCM β-d-glucoside (**5**, Figure 1; ≥99%) were provided by BASF. Note: 15HCM is a racemic mixture of the (3*R*, 5*R*, 6*S*) and (3*S*, 5*S*, 6*R*) forms (Figure 1). 15HCM and the glycosylated derivatives thereof are toxic and irritant. Proper care was taken in their handling. Calf intestine phosphatase (CIP, 10 000 U/mL) was from New England Biolabs (Frankfurt am Main, Germany).

### Enzymes

*Bc*GT1 (GenBank accession number: KT821092)^30^ was produced in *Escherichia coli* BL21(DE3). The gene optimized for expression in *E. coli* (Supporting Information) was inserted in pET15b as expression plasmid. The construct used encodes the native *Bc*GT1 without purification tag. Cells were cultivated at 37 °C and 110 rpm in 1 L baffled shake flasks containing 250 mL of Luria-Bertani (LB) medium supplemented with 115 μg mL^–1^ ampicillin. At an optical density (600 nm) of ∼1.0, the cells were induced with 1 mM isopropyl β-d-1-thiogalactopyranoside and incubated at 18 °C for 20 h. Centrifuged cells (Sorvall RC-5B Superspeed Centrifuge; DuPont, Wilmington, DE; 4 °C and 4500*g* for 25 min) were suspended (0.31 g cells/mL) in sodium phosphate buffer (25 mM, pH 7.0) containing 5 mM dithiothreitol. The suspension was sonicated using Fisherbrand Model 505 Sonic Dismembrator (Waltham, MA; 10 s on, 20 s off, amplitude 30%, 6 min total on-time). Cell-free extract was cleared by centrifugation at 4500*g* and 4 °C for 60 min and filtration with a 1.2 μm filter. *Bc*GT1 was purified by ion-exchange chromatography with a HiTrap DEAE FF column (5 mL, Cytiva, Chicago, IL) at 4 °C. The column was equilibrated in 25 mM sodium phosphate buffer (pH 7.0) containing 5 mM dithiothreitol, and 5–10 mL of cell extract (900–2000 mg protein) was loaded. *Bc*GT1 was eluted at 0.9 mL/min in the same buffer containing 100 mM NaCl. Eluted fractions containing *Bc*GT1 were desalted and concentrated at 4 °C and 4500*g* in Vivaspin Turbo 15 PES tubes (10 kDa molecular mass cutoff, Sartorius, Göttingen, Germany). The enzyme solution (45–55 mg/mL, >90% purity) was stored in 25 mM sodium phosphate (pH 7.0) at −80 °C. The purified enzyme was stable for at least 2 months.

Sucrose synthase from soybean (*Glycine max*, *Gm*Susy) was produced and purified as described in earlier work.^32,33^ The enzyme is equipped with an N-terminal Strep-Tag II. Enzyme purity was confirmed by SDS PAGE. Its specific activity as isolated was 4.1 U/mg for sucrose cleavage and 3.5 U/mg for sucrose synthesis.

Protein was determined with a DeNovix DS-11+ spectrophotometer (DeNovix, Inc., Wilmington, DE). Molecular mass and molar extinction coefficients were calculated using the ProParam tool in ExPASy.

### Glycosylation Reactions

Reactions were performed at 0.3 mL total volume in Eppendorf tubes, using agitation at 400 rpm with Thermomixer Comfort (Eppendorf, Hamburg, Germany). The temperature was 37 °C. The assay/reaction conditions used (acceptor substrate, donor, buffer, and co-solvent concentration) are summarized in Table 1. Unless stated, HEPES buffer (100 mM, pH 7.4) containing 5 mM MgCl_2_ was used. Reactions were started by adding enzyme (0.5–5.0 mg/mL) to substrate preincubated at 37 °C for 2 min. To stop the reaction, ice-cold acetonitrile was added to sample (1:1, by volume) and incubation was done on ice for 10 min. Precipitated enzyme was filtered off, and the liquid was analyzed further. Samples were taken at suitable times to measure the initial reaction rates (≤1 h) and to determine the course of conversion (up to 24 h). Consumption of the acceptor substrate and formation of glycosylated products were measured by HPLC. Reactions were typically performed over 24 h with samples taken regularly. One unit of activity is the enzyme amount consuming 1 μmol acceptor/min under the specified conditions.

**Table 1:** Specific Activities of *Bc*GT1 in (De)glycosylation Reactions of 15HCM, 4MU, 4NP, and the Corresponding β-d-Glucosides^a^^,^^f^

substrate	donor	concentration (mM) donor/substrate	substrate consumption (nmol/(min mg))	mono-glucoside formation (nmol/(min mg))	disaccharide product formation (nmol/(min mg))	aglycone (re)formation (nmol/(min mg))
15HCM	UDP-glucose	0.5/10	34.9^b^	31.7^b^	0.24^b^	N.D.
		0.5/1	16.8	16.2	N.D.	N.D.
		1/1	31.6 (39.1)^b^	30.5(38.1)^b^	N.D.(2.3)^b^	N.D.
		2/1	49.2 (30.9)^b^	47.2 (30.2)^b^	2.1 (2.3)^b^	N.D.
		5/1	47.3	44	2.8	N.D.
15HCM-β-d-glucoside	UDP-glucose	2/1	4.6	N.D.	4.6	N.D.
	UDP	1/1(1/5)^d^	N.D. (N.D.)^d^	N.D. (N.D.)^d^	N.D. (N.D.)^d^	N.D. (N.D.)^d^
4MU	UDP-glucose	2/1	211.7	206.3	N.D.	0.081
4MU-β-d-glucoside	UDP-glucose	2/1	0.35 (0.16)^c^	N.D. (N.D.)^c^	0.15 (0.14)^c^	0.24 (0.075)^c^
	UDP	2/1 (1/1)^d^	1.25 (1.16)^d^	N.D. (N.D.)^d^	0.0025 (N.D.)^d^	1.11 (1.16)^d^
	none	none/1	0.37 (0.05)^e^	N.D. (N.D.)^e^	N.D. (N.D.)^e^	0.37 (0.05)^e^
4NP	UDP-glucose	2/1	10.1	9.4	0.8	0.5
4NP-β-d-glucoside	UDP-glucose	2/1	1.3 (0.8)^c^	N.D. (N.D.)^c^	0.9 (0.5)^c^	0.4 (0.3)^c^

a The specific activities (nmol/(min mg)) are from triplicate determinations (*N* = 3) and have standard errors of 10% or less of the reported mean value. Conversion data and product distributions are from a single experiment, confirmed in one biological replicate (*N* = 2).

b UDP-glucose was regenerated by *Gm*Susy (0.05 mg/mL, 0.21 U^b^; 0.1 mg/mL, 0.41 U^c^) and sucrose (100 mM). HEPES buffers (100 mM, pH 7.0 for 15HCM and pH 7.4 for 4MU-β-d-glucoside and 4NP-β-d-glucoside) containing 5 mM MgCl_2_ were used.

c UDP-glucose was regenerated by *Gm*Susy (0.05 mg/mL, 0.21 U^b^; 0.1 mg/mL, 0.41 U^c^) and sucrose (100 mM). HEPES buffers (100 mM, pH 7.0 for 15HCM and pH 7.4 for 4MU-β-d-glucoside and 4NP-β-d-glucoside) containing 5 mM MgCl_2_ were used.

d Data were obtained with *Gm*Susy (0.1 mg/mL, 0.35 U) and fructose (100 mM) for UDP regeneration. Tris-HCl buffer (50 mM, pH 9.0) containing 5 mM MgCl_2_ was used. DMSO concentration was 4%. *Bc*GT1 was 1 mg/mL for 15HCM-β-d-glucoside and 3 mg/mL for 4MU-β-d-glucoside.

e To remove nucleotide diphosphate that co-purified with *Bc*GT1, the enzyme (5 mg/mL, 0.11 mM) was incubated with calf intestine phosphatase (20 U, 2 μL) for 30 min at 37 °C. Sodium phosphate buffer (100 mM, pH 7.4) containing 5 mM MgCl_2_ was used. Reaction was initiated by the addition of 4MU-β-d-glucoside (4% DMSO).

f N.D., not detectable.

### Reversed-Phase HPLC Analytics

Samples were analyzed on an Agilent 1200 HPLC system (Santa Clara, CA) equipped with a Kinetex EVO C18 column (5 μm, 100 Å, 150 × 4.6 mm; Phenomenex, Aschaffenburg, Germany) and a UV–vis detector. The column temperature was 45 °C. The injection volume was 5–10 μL. The eluent flow rate was 1 mL/min. The column was equilibrated in water containing 0.1% formic acid. Elution was done with an increasing gradient in acetonitrile containing 0.1% formic acid, starting from 5%.

#### Reactions with 15HCM and 15HCM-β-d-Glucoside

A gradient of 20–75% acetonitrile over 5.5 min was used. The column was washed with 75% acetonitrile for 2 min and equilibrated with 20% acetonitrile for 4.5 min. 15HCM and its mono- and di-β-d-glucosides were detected at 203 nm. Additionally, for the separation of 15HCM di- and tri-β-d-glucosides, isocratic method (20% acetonitrile for 20 min and 25% acetonitrile for 10 min) and gradient method (20–30% of acetonitrile for 40 min) were used. The column was washed with 30% acetonitrile for 3 min and equilibrated with 20% acetonitrile for 3 min. The product separation is shown in the Results and Discussion section (see also Supporting Figure S1).

#### Reactions with 4MU and 4MU-β-d-Glucoside

A gradient of 10–60% acetonitrile over 15 min was used. The column was washed with 90% acetonitrile for 2 min and equilibrated with 10% acetonitrile for 3 min. The 4MU and its β-d-glucosides were detected at 220 and 320 nm.

#### Reactions with 4NP and 4NP-β-d-Glucoside

A gradient of 5–60% acetonitrile over 15 min was used. The column was washed with 60% acetonitrile for 2 min and equilibrated with 5% acetonitrile for 3 min. 4NP and 4NP-β-d-glucoside were detected at 220 and 405 nm, respectively.

### Isolation of Disaccharide-Modified 15HCM

**Figure 2:**
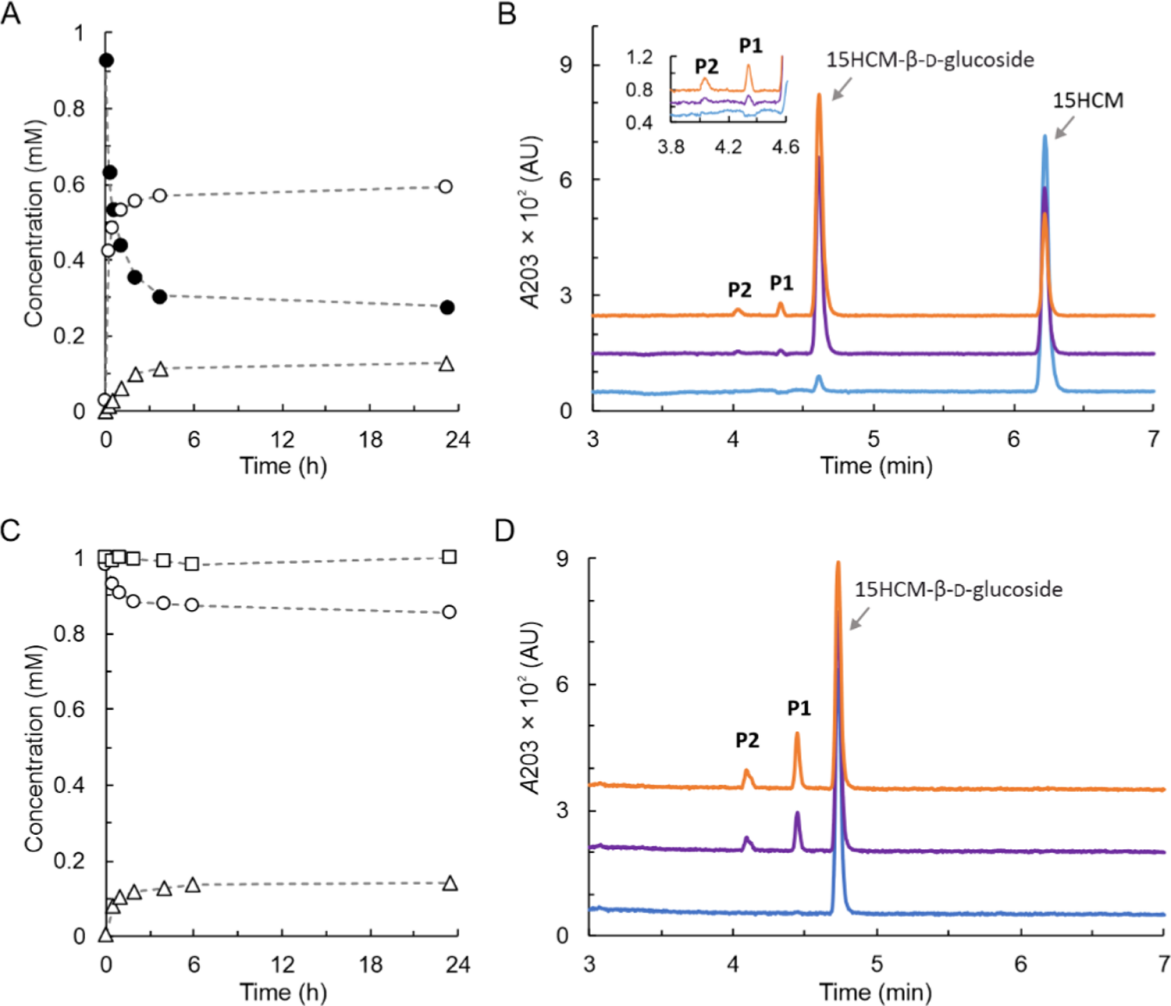
Enzymatic glycosylation of 15HCM and 15HCM-β-d-glucoside by *Bc*GT1. Reactions of 15HCM (A, B) and 15HCM-β-d-glucoside (C, D) with *Bc*GT1 (0.5 mg/mL) and UDP-glucose are shown. Initial rates are shown in Table 1. (A) Glycosylation of 15HCM (closed circles). Products were 15HCM-β-d-glucoside (open circles) and disaccharides (open triangles). Disaccharides were obtained as the sum of **P1** and **P2** shown in (B). (B) HPLC traces of glycosylation of 15HCM (0 h, blue; 0.5 h, purple; 3.7 h, orange). The inset is the close-up of disaccharides (**P1**, 4.3 ± 0.2 min; **P2**, 4.0 ± 0.1 min) formed by *Bc*GT1. (C) Glycosylation of 15HCM-β-d-glucoside (open circles) to disaccharides (open triangles). Disaccharides were obtained as the sum of **P1** and **P2**, as shown in (D). Reaction of 15HCM-β-d-glucoside lacking *Bc*GT1 is shown in open squares. (D) HPLC traces of glycosylation of 15HCM-β-d-glucoside (0 h, blue; 0.5 h, purple; and 2 h, orange).

The glycosylated products of 15HCM-β-d-glucoside (Figure 2, **P1** and **P2**) were isolated from the enzymatic reaction, and their chemical identities were determined from MS and NMR analyses. Synthesis was performed with *Bc*GT1 (1 mg/mL) using 15HCM-β-d-glucoside (5 mM) in water. UDP-glucose was supplied *in situ* from UDP (1 mM) and sucrose (100 mM) using *Gm*Susy (0.1 mg/mL, 0.41 U). The total volume was 0.9 mL. The temperature was 37 °C. After 24 h, 15HCM-β-d-glucoside was consumed up to 50% (∼2.5 mM). The enzymes were filtered off. The products were purified by reversed-phase HPLC using the Kinetex EVO C18 column used also for analytical determinations. Pooled fractions (25 mL) were concentrated to about one-twentieth the original volume using a Heidolph Laborota 4000 rotary evaporator equipped with a vacuubrand PC2001 pump and CVC2000II controller (Wertheim, Germany; 40 °C, <230 mbar) and then lyophilized overnight with a freeze dryer Alpha 1-4 (Martin Christ Gefriertrocknungsanlagen GmbH, Osterode am Harz, Germany) at −40 °C and 0.020 mbar. The isolated products were used for MS and NMR analyses.

### MS Analysis

The glycosylated products from the reaction of *Bc*GT1 with 15HCM-β-d-glucoside were analyzed. An UltiMate3000 system (Thermo Fisher Scientific, MA) equipped with a Luna C18 column (5 μm, 100 Å, 3.0 × 250 mm, Phenomenex, CA) and a high-resolution mass spectrometer (Q Exactive Hybrid Quadrupole-Orbitrap, Thermo Fisher Scientific) was used. A gradient (5 to 100%) of acetonitrile in 5 mM ammonium acetate buffer (pH ∼ 7) over 5 min was used. The column was washed with 100% acetonitrile for 5 min and then 5% acetonitrile for 25 min. The flow rate was 1 mL/min. The column temperature was 30 °C. The UV–vis detector was set to scan the range 190–450 nm. Masses were scanned over the range of *m*/*z* 70–1000 with positive/negative switching electrospray ionization mode. The masses of *bis*-glucosides (614.3; [M + H]^+^, 615.3; [M + NH_4_]^+^, 632.3; [M – H]^−^, 613.3; [M + CH_3_COO]^−^, 673.3) and *tri*-glucosides (776.4; [M + H]^+^, 777.3; [M + NH_4_]^+^, 794.4; [M – H]^−^, 775.3; [M + CH_3_COO]^−^, 835.4) were also analyzed in extracted-ion chromatogram. The obtained data are shown in Supporting Figures S2 and S3.

### NMR

The disaccharide-modified 15HCM isolated from the reaction with 15HCM-β-d-glucoside was analyzed. The products were taken up in DMSO-d_6_ (99.8% D) for NMR measurements. A Bruker Avance Neo 700 spectrometer equipped with a TCI-cryoprobe (Bruker Biospin, Rheinstetten, Germany) and Spectrus processor 2018.2 software (ACD Labs, Toronto, Canada) were used at 298 K. ^1^H- and ^13^C NMR, HSQC–DEPT, COSY, and HMBC spectra were recorded. The chemical shifts of DMSO-D_6_ were 2.50 and 39.52 ppm. The obtained data are shown in Supporting Tables S1 and S2 and Figures S4–S9.

### Protein Structural Modeling and Docking

The PyMOL molecular graphics system (incentive version; Schrödinger, LLC) was used for structural alignments. Structure modeling and docking were performed with YASARA v. 18.2.7 (Yasara Biosciences GmbH, Vienna, Austria). *Bc*GT1 model was built upon the crystal structure of CalG2 (calicheamicin glycosyltransferase) bound with thymidine-5′-diphosphate and calicheamicin T0 (PDB accession number 3RSC). YASARA’s homology modeling macro was used. All ligands (15HCM β-d-glucosyl-(1→4)-β-d-glucoside, 15HCM β-d-glucosyl-(1→6)-β-d-glucoside, UDP-glucose) were generated using the Grade Web Server (http://grade.globalphasing.org/cgi-bin/grade/server.cgi). Local ligand docking was carried out with Autodock Vina^34^ using standard parameters. Following the docking runs, the obtained poses were optimized by energy using the standard macro included in YASARA, except that the number of runs was increased to 50. Similar docking poses (<5.0 Å RMSD in superposition based on the protein structure) were clustered. For dockings of β-d-cellobiosyl- and β-d-gentiobiosyl-15HCM, the calicheamicin T0 was deleted and a simulation cell of 15 Å × 10 Å × 10 Å was placed at the former location. The same procedure was used for docking of UDP-glucose, except that thymidine-5′-diphosphate was removed.

Docking poses/clusters were evaluated by their associated free energy and mechanistic plausibility. The protonation states of all other protein residues were set automatically for a pH of 7.00. The calculated binding energies of β-d-cellobiosyl- and β-d-gentiobiosyl-15HCM were 7.9 ± 0.3 (*n* = 10) and 9.2 ± 0.3 (*n* = 5) kcal mol^–1^, respectively, corresponding to dissociation constants of 1.6 and 0.2 μM. The binding energy of UDP-glucose was 7.9 ± 1.0 (*n* = 9) kcal mol^–1^, corresponding to a dissociation constant of 1.7 μM. The *Z*-score for the homology model of *Bc*GT1 was −1.94.

## Results and Discussion

### Iterative Glycosylation of 15HCM

Time course of the *Bc*GT1 reaction with 15HCM (1.0 mM; 4% DMSO, by volume) and UDP-glucose (2.0 mM) is shown in Figure 2A. The 15HCM was converted rapidly to ∼44% within about 1 h. This corresponded to a specific activity of *Bc*GT1 of 49.2 nmol/(min mg). The reaction slowed down later and leveled out at ∼73% conversion of the initial 15HCM. The 15HCM β-d-glucoside was the main product, formed in ∼60% of the 15HCM converted. The remainder ∼13% of the 15HCM converted was a mixture of likely disaccharide-modified products of the 15HCM, as follows. Figure 2B shows a superimposition of HPLC traces of samples from the reaction. Besides the 15HCM β-d-glucoside peak, two additional peaks, labeled **P1** and **P2** in the HPLC trace, emerged in correspondence to the reaction progress.

As judged from their elution times, the two peaks represent compounds more polar than the 15HCM β-d-glucoside. HPLC with ESI-MS detection showed the same primary mass for the main constituent in each peak (614; 615, [M + H]^+^; 637.6, [M + Na]^+^; 653.6, [M + K]^+^), corresponding to 15HCM with two glucosyl residues attached.^31^ From their corresponding peak areas, **P1** and **P2** were present in a ratio of approximately 2:1. The ratio of the two peaks did not change, dependent on conditions varied or variable, including the enzyme concentration used, the degree of 15HCM conversion, or the overall amount of **P1** and **P2** formed.

The implied reaction sequence, 15HCM → 15HCM β-d-glucoside → 15HCM β-d-glucosyl-β-d-glucoside, was examined in an experiment that offered 15HCM β-d-glucoside (1.0 mM) as enzyme substrate for glycosylation from UDP-glucose (2.0 mM). The time course of 15HCM β-d-glucoside consumption is shown in Figure 2C. The same peaks **P1** and **P2** observed from the enzymatic reaction of 15HCM (Figure 2B) were formed in identical 2:1 ratio in correspondence to the usage of the 15HCM β-d-glucoside (Figure 2D). From the data in Figure 2C at times ≤2 h, the specific *Bc*GT1 activity for glycosylation of 15HCM β-d-glucoside was determined as 4.6 nmol/(min mg) (Table 1). A roughly similar specific activity of ∼2.3 nmol/(min mg) was estimated from Figure 2A for the formation of the proposed 15HCM β-d-glucosyl- β-d-glucosides (sum of **P1** and **P2**). These specific activities were lower by about 10-fold than the specific *Bc*GT1 activity for 15HCM β-d-glucoside formation of 47.2 nmol/(min mg).

### Identification and Structural Characterization of Disaccharide-Modified 15HCM

**Figure 3:**
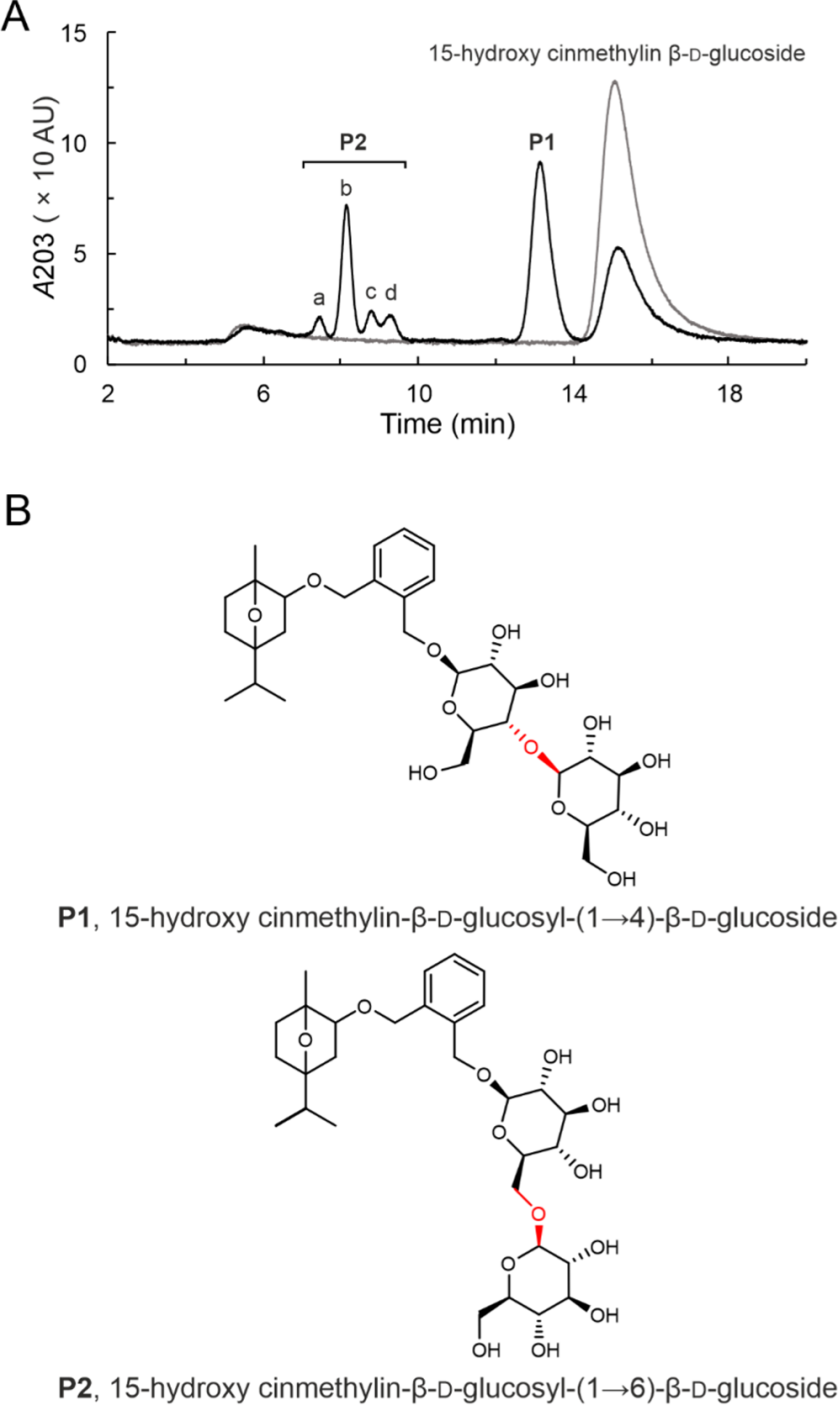
Disaccharide products from the reaction of 15HCM-β-d-glucoside with *Bc*GT1. (A) HPLC traces of product separation. The isocratic method (20% of acetonitrile in water, both solvents contain 0.1% formic acid) was used. 15HCM-β-d-glucoside (gray line) was eluted at 15 min. After incubation for 23 h (black line), 15HCM-β-d-glucoside was converted to **P1** (13.1 min) and **P2** (7.5–9.3 min; a, 7.5 min; b, 8.1 min; c, 8.8 min; d, 9.3 min). (B) Chemical structures of product **P1** (15HCM-β-d-cellobioside) and **P2** (15HCM-β-d-gentiobioside). The products were identified by MS and NMR analysis, as shown in Supporting Tables S1 and S2 and Supporting Figures S2–S9. The a, c, and d of **P2** are trisaccharide-modified products of 15HCM as shown in Supporting Figure S3.

Products of iterative glycosylation of 15HCM were isolated by preparative HPLC, using the same column as applied in the analytical determinations. Different gradient methods were used in an effort to optimize the separation (Figure 3 and Supporting Figure S1). Using isocratic elution with 20% acetonitrile (Figure 3), peak **P1** was not further separated and appeared to be from a single compound. Peak **P2** was separated into one major and three smaller peaks, as shown in Figure 3, suggesting a complex composition. Isolated peaks **P1** (∼0.73 mg) and **P2** (fractions a–d; ∼0.33 mg) were characterized by high-resolution NMR and MS. The results are shown in Figure 3, with additional data shown in Supporting Figures S2–S9 and Tables S1 and S2. Peak **P1** (Figure 3) was assigned unambiguously the chemical structure of 15HCM β-d-glucosyl-(1→4)-β-d-glucoside (Supporting Table S1, Supporting Figures S2, S4–S8). The primary glycosidic linkage with the hydroxy group of 15HCM was indicated by the correlation of C22 (CH, 4.25 ppm, 101.88 ppm) with C15 (CH_2_, 4.62 ppm and 4.86 ppm, 67.45 ppm), as shown in Supporting Figure S8. Iterative glycosylation was observed on C25 (CH, 3.34 ppm, 80.57 ppm) of the primary glucosyl residue showing correlation with C33 (CH, 4.26 ppm, 103.22 ppm) of the secondary glucosyl residue, clearly indicating a β(1→4) bond (Supporting Figure S8). Additionally, proton and carbon peaks of C22 of the diastereomeric structures of 15HCM β-d-glucosyl-(1→4)-β-d-glucoside were observed at a ratio of approximately 1:1 (Supporting Figures S4 and S5). By contrast, the proton and carbon peaks of C33 of the diastereomeric structures were hardly observed, plausibly because of their considerable distance from the chiral center C5 (Supporting Figures S4 and S5). Peak **P2** was shown to contain 15HCM β-d-glucosyl-(1→6)-β-d-glucoside (Figure 3) as the major component (∼85%; fraction b; Supporting Table S2, Supporting Figures S3 and S9), as revealed by NMR and MS. Three trisaccharide glycosides of 15HCM were additionally present (total ∼15%; fractions a, c, and d; MS data in Supporting Figure S3). Despite the mixture, the glycosidic linkages of the disaccharide-modified 15HCM were clearly identified from 2D-NMR spectra (Supporting Figure S9). Relevant signals from C22 (CH, 4.19 ppm, 102.22 ppm), C33 (CH, 4.30 ppm, 103.79 ppm), and C29 (CH_2_, 3.60 ppm/4.01 ppm, 68.98 ppm) were revealed in the HSQC spectra (Supporting Figure S9B,C). In HMBC spectra, correlation of the C22 proton and the C15 carbon identified the glycoside on 15HCM (Supporting Figure S9D), while the correlation of the C33 proton and the C29 carbon showed the disaccharide (Supporting Figure S9D). Additionally, correlation between the two C29 protons and the C33 carbon was shown (Supporting Figure S9E). From these results, and considering the strict β stereoselectivity of the enzyme in glycosylation, the formed disaccharide linkage was assignable clearly as β(1→6), implying a β-gentiobiosyl unit attached to 15HCM.

An interesting NMR observation was that in the β(1→6) disaccharide product, both anomeric glucosyl carbons (C22, C33) showed double doublets (Supporting Figure S9B). In the β(1→4) disaccharide product, by contrast, only the C22 gave a double doublet while the C33 signal was a single doublet. Considering that the 15HCM used was a mixture of two stereoisomers, the double doublet was plausibly explained as diastereomeric split of signal for the β-glucosyl carbon (C22) immediately attached to the isomeric 15HCM. Diastereomeric split for C33 in the β-gentiobiosyl product can probably be attributed to through-space interactions with the 15HCM moiety enabled by the relatively high torsional mobility of the β(1→6) linked D-glucosyl residue. Lacking rotational freedom in the β-cellobiosyl product, analogous through-space interactions are not possible for the β(1→4) linked glucosyl residue and diastereomeric split of C33 signal is therefore not observed. Our interpretation is consistent with the literature showing diastereomeric split of signal for the anomeric proton in the distal sugar of a β(1→6) disaccharide glycoside, (7*S*)- and (7*R*)-phenylcyanomethyl 1′-*O*-α-L-rhamnosyl-(1→6)-β-d-glucosides.^35^

### Thermodynamic and Kinetic Requirements for Iterative Glycosylation of 15HCM

**Figure 4:**
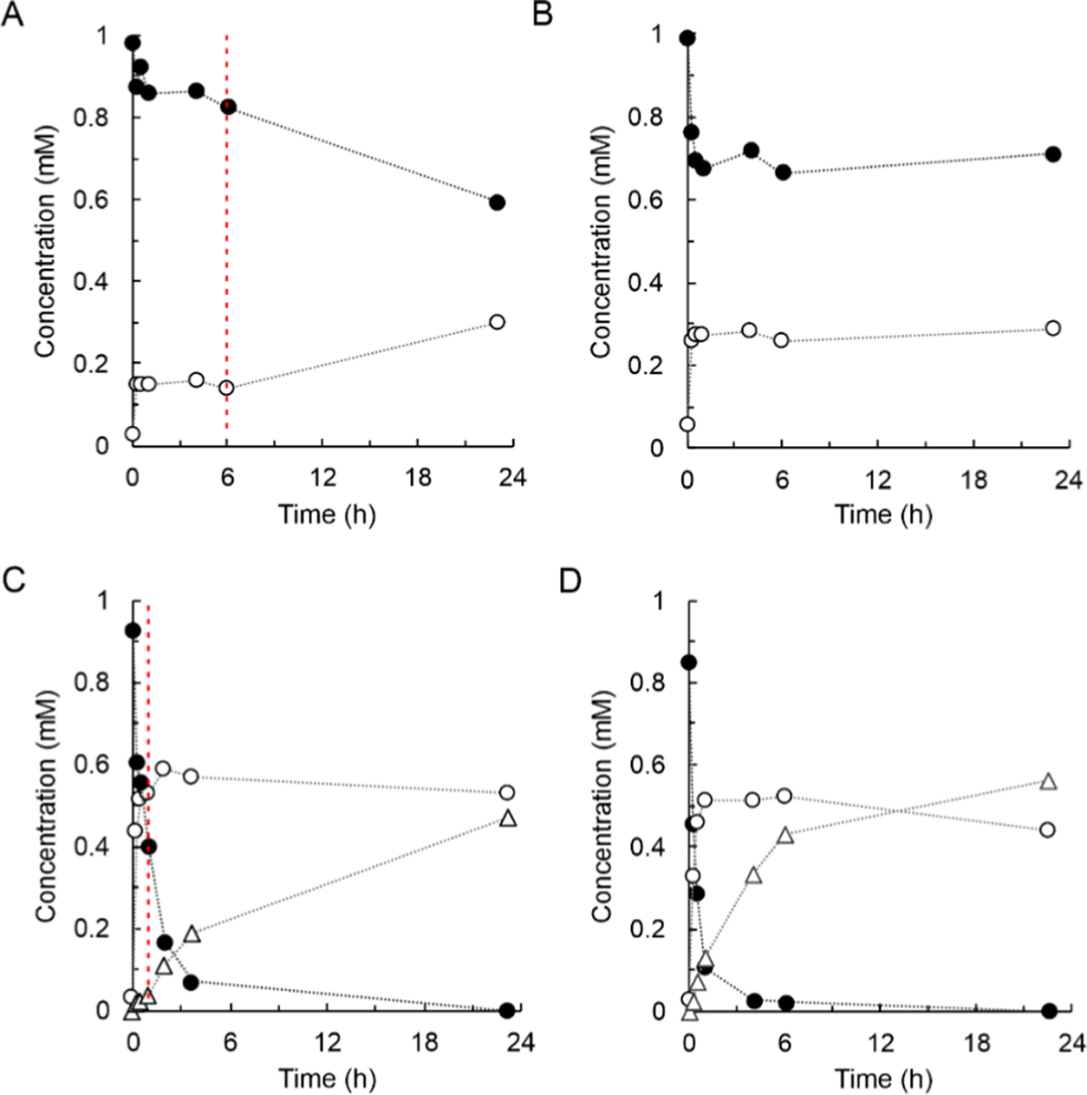
Effects of UDP-glucose concentration on enzymatic glycosylation of 15HCM by *Bc*GT1. 15HCM (closed circles) was used with DMSO (4%, v/v) as co-solvent. *Bc*GT1 was 0.5 mg/mL. UDP-glucose concentration was 0.5 mM (A), 1 mM (B), 2 mM (C), and 5 mM (D). Red dotted lines indicate secondary addition of UDP-glucose (A, 0.5 mM; C, 2 mM). Products are 15HCM-β-d-glucoside (open circles) and disaccharides (open triangles). Disaccharides were obtained as a sum of **P1** and **P2**, as shown in Figure 2. Initial rates are shown in Table 1.

Enzymatic reactions were performed in which the UDP-glucose concentration (0.5–5.0 mM) was varied at a constant concentration of 15HCM (1.0 mM). Figure 4 shows the results. The 15HCM conversion increased linearly depending on the UDP-glucose supplied, consistent with the effect of mass action. The experiment in which 1.0 mM UDP-glucose was used at once (Figure 4B) or was added in two portions of 0.5 mM (Figure 4A) gave the same 15HCM conversion due to the formation of 15HCM β-d-glucoside as the sole product. An equilibrium constant of ∼40 (=[15HCM-β-d-glucoside][UDP]/[15HCM][UDP-glucose]) could be estimated from the concentration ratios of substrates and products at apparent equilibrium for the single-step glycosylation reaction (after 24 h), 15HCM + UDP-glucose ↔ 15HCM β-d-glucoside + UDP.

Excess of UDP-glucose over 15HCM was important for the formation of the disaccharide-modified 15HCM, as shown in Figures 2A and 4C–D. Using UDP-glucose in twofold excess (2.0 mM; Figure 2A), the disaccharide glucosides were released at ∼0.13 mM, representing ∼60% of the total 15HCM glucosides formed under these conditions. The 15HCM conversion was 72%. Experiment in which UDP-glucose was added in two portions of 2.0 mM, as shown in Figure 4C, revealed iteratively glycosylated products (0.47 mM) released in the same amount overall as the 15HCM β-d-glucoside (0.53 mM). The 15HCM was converted completely. Interestingly, the concentration of 15HCM β-d-glucoside hardly changed after the UDP-glucose addition, despite the fact that ∼40% 15HCM substrate was still available at the time. The disaccharide-modified products were formed in a larger amount only after UDP-glucose addition. Dynamic equilibrium between singly and iteratively glycosylated products of the 15HCM was suggested. Effect of the fresh UDP-glucose was to shift the equilibrium ratio between the two products, with the net result of accumulation solely of the disaccharide-modified 15HCM (Figure 4C).

Using 5.0 mM UDP-glucose, the 15HCM was converted fully in a single-batch reaction (Figure 4D). The disaccharide-modified products accumulated in an overall concentration (0.56 mM) slightly exceeding that of the 15HCM β-d-glucoside (0.44 mM). Kinetically, the disaccharide glucosides continued to be formed after the point of 15HCM β-d-glucoside released at maximum concentration (∼0.5 mM). The results show that even at a high ratio of UDP-glucose/15HCM, the enzymatic glycosylation yielded a mixture of singly and iteratively glycosylated products. Initial rate analysis indicated that further increase in donor/acceptor ratio resulting from an increase in the UDP-glucose concentration would likely not be efficient. Specific *Bc*GT1 activities of 15HCM β-d-glucoside formation of 16 nmol/(min mg), 31 nmol/(min mg), 47 nmol/(min mg), and 44 nmol/(min mg) were determined for reactions at 0.5 mM (Figure 4A), 1.0 mM (Figure 4B), 2.0 mM (Figures 2A and 4C), and 5.0 mM UDP-glucose (Figure 4D), respectively.

### Disaccharide Modification of 15HCM with Product Control

**Figure 5:**
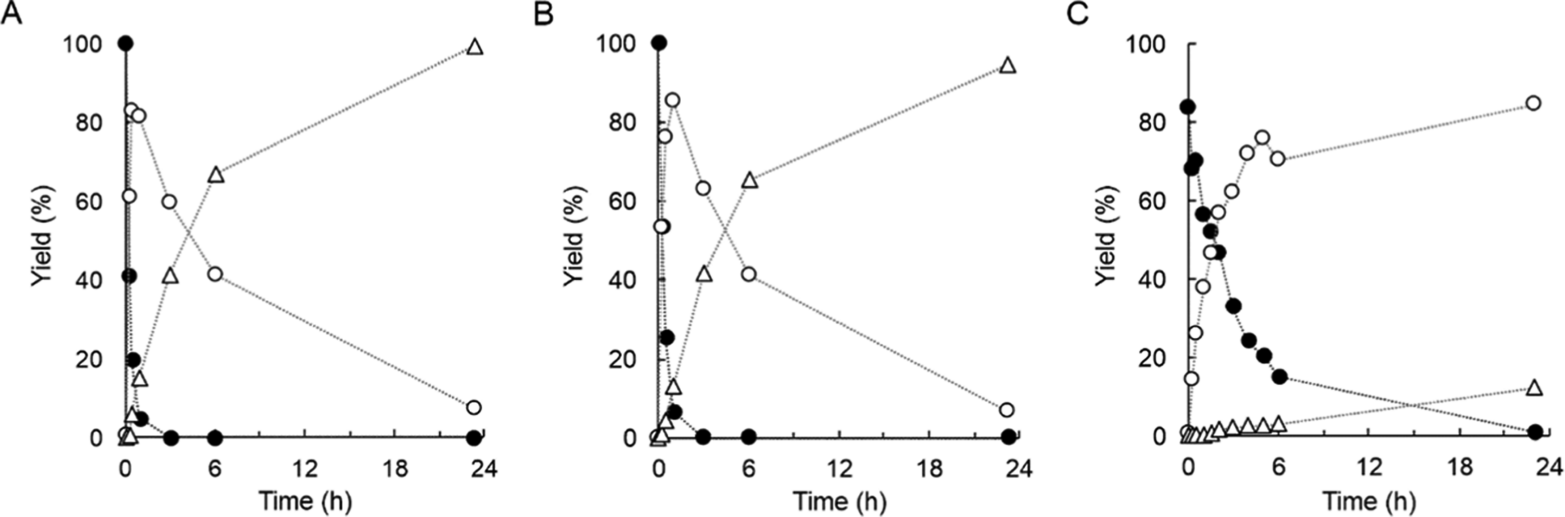
Enzymatic glycosylation of 15HCM with UDP-glucose regeneration. UDP-glucose was regenerated from sucrose conversion by *Gm*Susy and UDP. 15HCM (A, B, 1 mM; C, 10 mM) was used. DMSO (A, B, 4%; C, 10%) was used as a co-solvent. UDP concentrations were 1 mM (A), 2 mM (B), and 0.5 mM (C). Closed circles are 15HCM. Open circles are 15HCM-β-d-glucoside. Open triangles are disaccharide forms of 15HCM, a sum of **P1** and **P2** as shown in Figure 2. Initial rates are shown in Table 1.

To obtain better control over the glycosylated products formed from 15HCM than was possible by varying the donor/acceptor ratio with UDP-glucose, we turned to a coupled reaction of *Bc*GT1 and sucrose synthase, with the idea of supplying the UDP-glucose donor *in situ* from sucrose and UDP.^36−39^ We considered that the sucrose synthase reaction might enable a constant steady-state level of UDP-glucose during the synthesis, contrary to the continuously changing conditions when UDP-glucose is used directly. We were pleased to discover conditions achieving selective production of the disaccharide-modified product at full conversion of the 15HCM substrate, as shown in Figure 5.

The overall reaction involved rapid formation of 15HCM β-d-glucoside in a yield of ∼85% of the 15HCM present (Figure 5A,B). The 15HCM β-d-glucoside was then gradually converted into the disaccharide glucosides (Figure 5A,B). Product selectivity was tunable via the UDP concentration used, where high and low concentrations led to the disaccharide glucosides (≥90% selectivity; Figure 5A–B) and formation of 15HCM β-d-glucoside (≥85% selectivity; Figure 5C). To our knowledge, this is the first report of selectivity in glycosyltransferase-catalyzed glycosylation modulated via *in situ* supply of the sugar nucleotide donor. As a general reaction concept, the approach seems to be broadly applicable to glycosyltransferases, but its relevance is immediately evident for glycosylation processes that are of an iterative nature. Adjustment of the steady-state level of sugar nucleotide donor via the nucleotide diphosphate concentration enables control over product formed from single or multiple events of glycosylation. The type of control is largely thermodynamic. Kinetic benefit may arise from lowered inhibition by the released nucleotide diphosphate. The permissive character of the sucrose synthase allows for different nucleotide-activated forms of d-glucose to be prepared from sucrose.^36,40,41^ Combination with a suitable sugar nucleotide epimerase can expand the scope of glycosyl donors prepared *in situ*.^36,42^ Other “reverse” glycosyltransferase reactions previously applied for *in situ* release of sugar nucleotide donors^10,43^ can be used in a conceptually analogous manner. Difference should be noted between the idea of *in situ* sugar nucleotide synthesis for controlling the product selectivity in iterative glycosylation processes, which is new, and use of the same for a mere regeneration of the sugar nucleotide, which is well established for biocatalytic synthesis with glycosyltransferases.^37−39^

### Glycosylation of 4MU and 4NP: Complex Interplay of Kinetics and Thermodynamics of *Bc*GT1-Catalyzed Reactions

**Figure 6:**
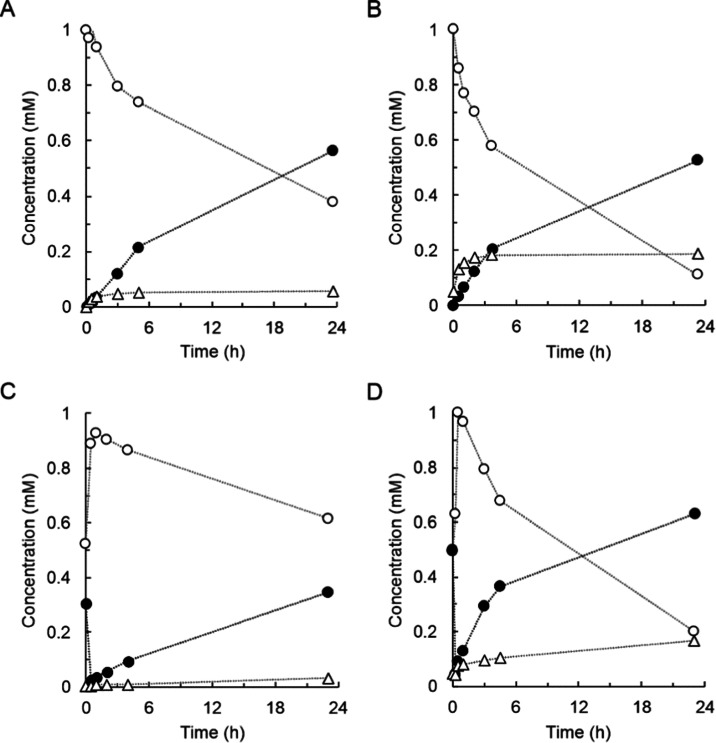
Reactions of 4MU-β-d-glucoside, 4NP-β-d-glucoside, and aglycones with *Bc*GT1 and UDP-glucose. Glucosides (open circles) and aglycones (closed circles) were 1 mM in the presence of DMSO (4%, by volume). *Bc*GT1 was 3 mg/mL. UDP-glucose was 2 mM. Open triangles are disaccharides. Initial rates are shown in Table 1. (A) Reaction of 4MU-β-d-glucoside. (B) Reaction of 4NP-β-d-glucoside. (C) Reaction of 4MU. (D) Reaction of 4NP.

To examine disaccharide modification of other aglycones, we used 4MU and 4NP, which are both broadly important xenobiotics (e.g., due to widespread use in analytical reagents). Despite earlier evidence of *Bc*GT1 glycosylating these acceptors,^30^ the reactions were not analyzed in detail. We first analyzed the *Bc*GT1 reaction with the β-d-glucosides of 4MU and 4NP. Results in Figure 6A,B reveal the glycosylation of both substrates from UDP-glucose, with a specific activity of 0.35 nmol/(min mg) (4MU β-d-glucoside) and 1.29 nmol/(min mg) (4NP β-d-glucoside) calculated from the data. Compared to the reaction of 15HCM β-d-glucoside that was cleanly converted to the disaccharide products (Figure 2C), the reactions of 4MU β-d-glucoside and 4NP β-d-glucoside were more complex in that besides the anticipated disaccharide products, free aglycone was also released. Glycosylation of 4MU or 4NP from UDP-glucose proceeded to full conversion of the aglycone, only to release free 4MU or 4NP later in the reaction (Figure 6C,D). These results suggested that the equilibrium reaction, aglycone + UDP-glucose ↔ β-d-glycopyranoside + UDP, was overlapped with another reaction perturbing the equilibrium position.

**Figure 7:**
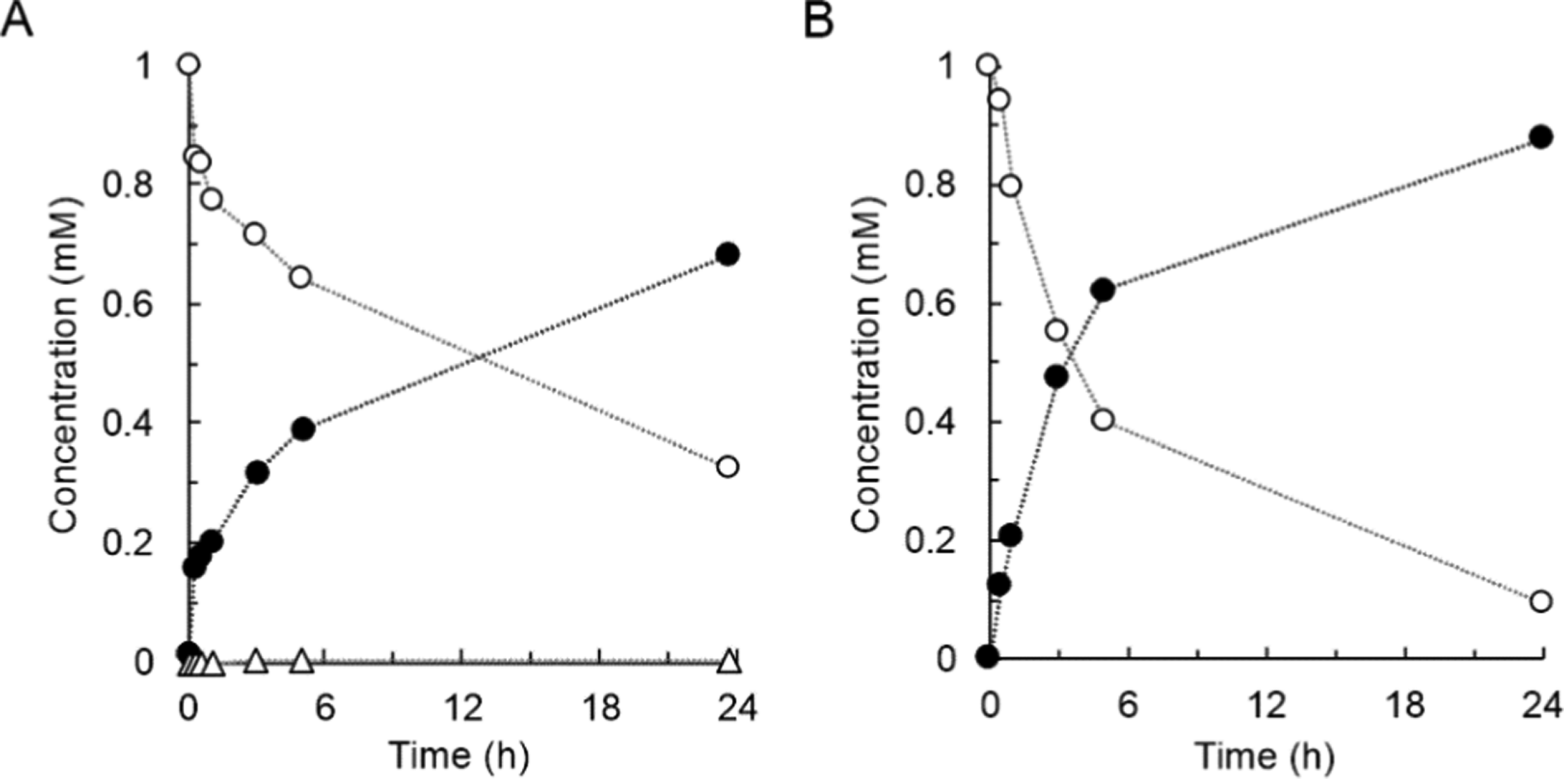
De-glycosylation of 4MU-β-d-glucoside by *Bc*GT1 and UDP. The enzyme *Bc*GT1 (3 mg/mL) catalyzes the transfer of β-glucosyl from 4MU-β-d-glucoside (1 mM, open circles) to 4MU (closed circles). Formation of disaccharides (open triangles) was not observed. (A) Reaction with UDP (2 mM) at pH 7.4. (B) Reaction with UDP regenerated from sucrose synthesis by *Gm*Susy with fructose and UDP-glucose. Initial rates are shown in Table 1.

The rate of re-formation of 4MU was dependent on the *Bc*GT1 concentration (Supporting Figure S10). An apparent specific activity of 0.081 nmol/(min mg) was determined for the enzyme preparation used. To exclude the possibility that the *Bc*GT1 as isolated contained the activity of a 4MU β-d-glucoside hydrolase, likely indicating a contamination, we offered 4MU β-d-glucoside as the sole substrate, without UDP-glucose or UDP added (Supporting Figure S11). The enzyme converted a limited amount of the substrate (∼0.25 mM) to 4MU, roughly corresponding to the molarity of the *Bc*GT1 used (Supporting Figure S11). Since this behavior was unusual for a hydrolase, we considered that nucleotide diphosphate co-purified with the *Bc*GT1 might promote glycosyltransferase reaction in the reverse direction. Indeed, *Bc*GT1 preparation stripped of nucleotide diphosphate by treatment with phosphatase^44,45^ no longer showed activity (Supporting Figure S11). De-glycosylation of 4MU β-d-glucoside to a nucleotide diphosphate acceptor, suggested from these findings, was further demonstrated in *Bc*GT1 reactions that involved 4MU β-d-glucoside and UDP as the substrates and showed conversion into 4MU and UDP-glucose (Figure 7A). The same conversion was shown with UDP supplied *in situ* from UDP-glucose and fructose via the reverse reaction of sucrose synthase (Figure 7B). Interestingly, no enzyme-catalyzed de-glycosylation was shown for the 15HCM β-d-glucoside in the presence of UDP, directly added to the reaction or released *in situ* (Supporting Figure S12). Considering apparent equilibrium of the 15HCM β-d-glucoside synthesis (Figure 4), restriction on the reverse reaction must be of a kinetic rather than thermodynamic nature.

With reversible glucosyl transfer between 4MU and UDP shown, it remained to explain that 4MU was slowly formed from a mixture in which the enzymatic reaction of 4MU and UDP-glucose had come to apparent equilibrium (Figure 6C). Considering that glycosyltransferases vary in degree to which they exhibit hydrolase activity toward their donor substrates,^4,46^ we examined the same for *Bc*GT1 and show hydrolysis of UDP-glucose by the enzyme (Supporting Figure S13). The specific activity was about 5% (∼10 nmol/(min mg)) that of the glycosyl transfer from UDP-glucose to 4MU (∼212 nmol/(min mg), Table 1).

### Controlled Glycosylation to Disaccharide-Modified 4MU and 4NP

**Figure 8:**
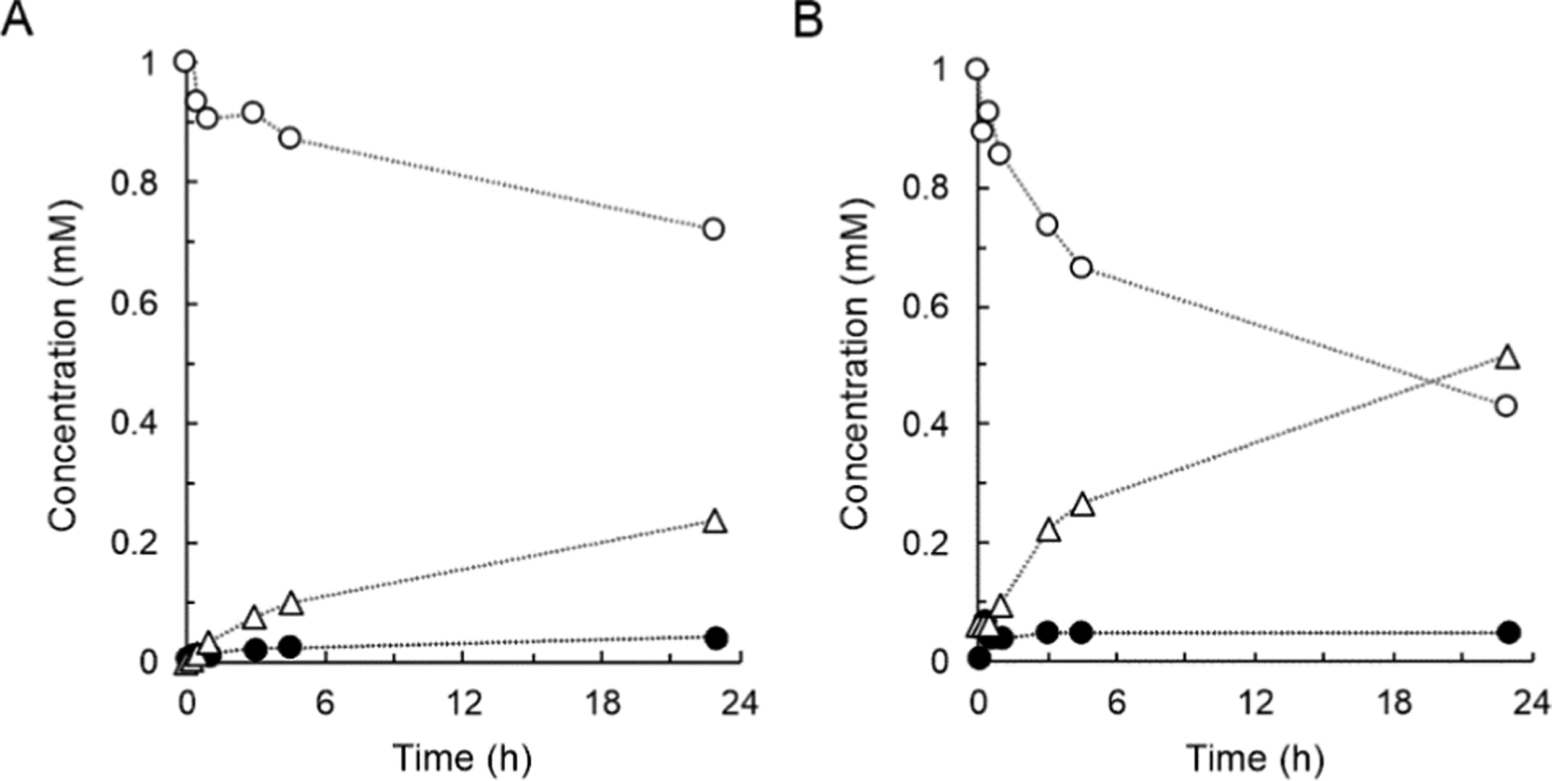
Glycosylation of 4MU-β-d-glucoside and 4NP-β-d-glucoside by *Bc*GT1 with enzymatic recycling of UDP-glucose. 4MU-β-d-glucoside (A, open circles) and 4NP-β-d-glucoside (B, open circles) concentrations were 1 mM. DMSO (4%, by volume) was used as a co-solvent. *Bc*GT1 concentration was 3 mg/mL. UDP-glucose was constantly supplied from the reaction of *Gm*Susy with sucrose and UDP. Closed circles are 4MU (A) and 4NP (B). Open triangles are disaccharide forms. Initial rates are shown in Table 1.

Being interested in synthetic access to disaccharide-modified 4MU and 4NP, we were encouraged by the results of 15HCM glycosylation (Figure 5) and considered glycosylation of the 4MU and 4NP mono-β-d-glucoside under conditions in which *in situ* release of UDP-glucose from sucrose and UDP controls the overall transformation. Using 2 mM UDP (as suggested from Figure 5B), our aim was to prevent the de-glycosylation of the mono-β-d-glucoside substrate by way of a relatively high steady-state concentration of UDP-glucose. Figure 8 shows that 4MU-β-d-glucoside (panel A) and 4NP-β-d-glucoside (panel B) both were converted with high selectivity (≥90%) for glycosylation compared to their degradation (de-glycosylation) into the corresponding aglycone. It should be instructive to compare *Bc*GT1 reactions with UDP-glucose added directly, leading mainly to de-glycosylation (Figure 6A,B), and *Bc*GT1 reactions with UDP-glucose released *in situ*, giving almost exclusively glycosylation (Figure 8).

Time courses in Figure 8 show steady formation of the disaccharide-modified products in high selectivity, with yields of 24% (4MU-β-d-glucoside) and 52% (4NP-β-d-glucoside) after 24 h. The yield can still be improved by extending the reaction time. The important point is the tight control of reaction direction achieved by *in situ* release of UDP-glucose. This control is absolutely crucial for the iterative glycosylation to become practical in providing synthetic access to disaccharide-modified aglycones. As already mentioned in discussing 15HCM glycosylation, the idea of directing iterative glycosylation reactions by way of delivering the sugar nucleotide donor could be broadly applicable to glycosyltransferases in synthesis.

### Permissive Nature of *Bc*GT1 Interpreted Structurally

**Figure 9:**
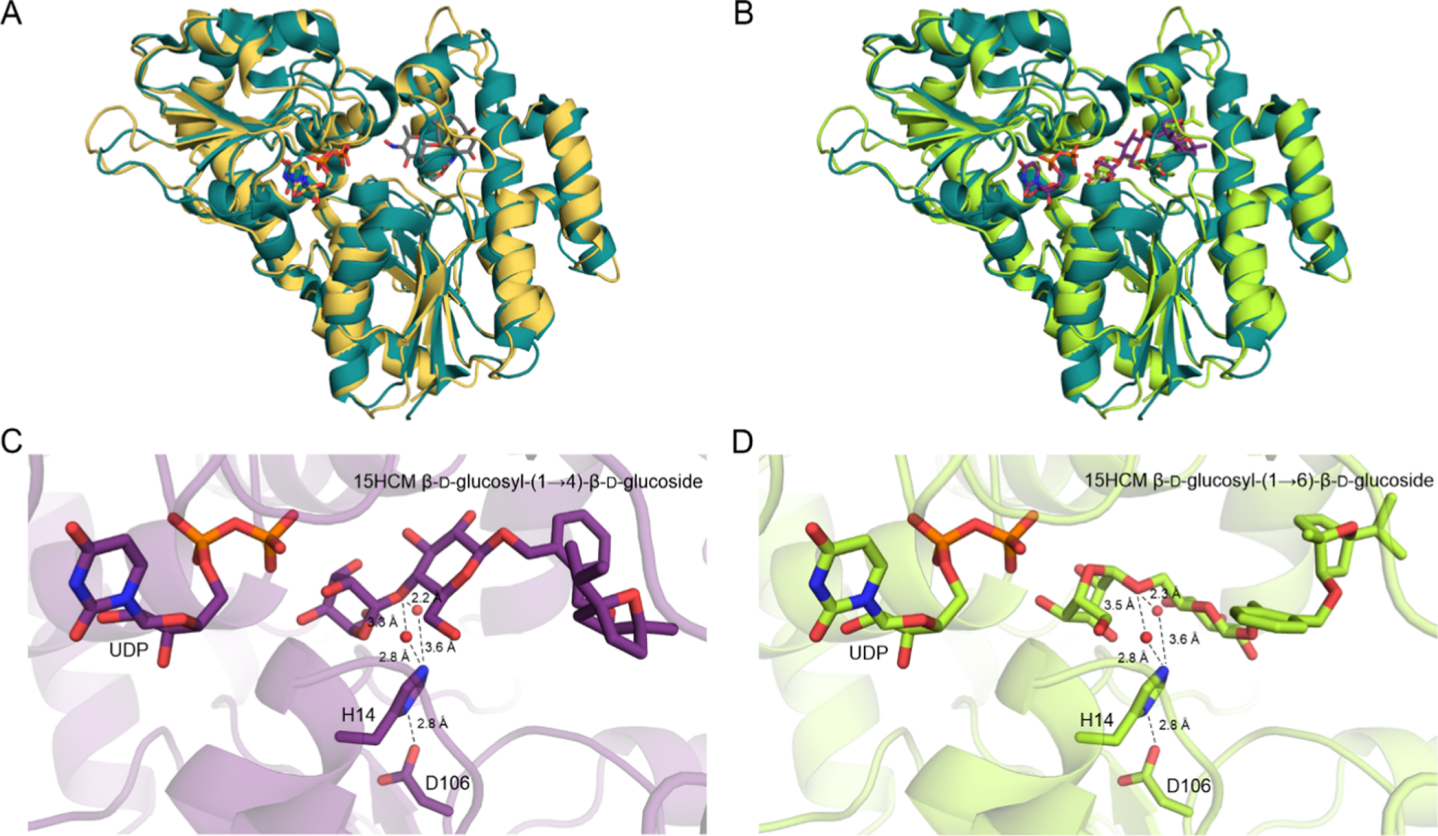
Structural interpretation of the iterative glycosylation of 15HCM-β-d-glucoside by *Bc*GT1. (A) Overlay of the structure model of *Bc*GT1 (UDP bound; protein and ligand shown in yellow-orange) and the crystal structure of CalG2 from *Micromonospora echinospora* (thymidine-5′-diphosphate and calicheamicin T0 bound, PDB code: 3RSC; protein and ligand shown in deep teal, calicheamicin T0 in gray). (B) Structure model of *Bc*GT1 with UDP and β-cellobiosyl 15HCM (shown in violet purple) or UDP and β-gentiobiosyl 15HCM (shown in lemon) bound. The crystal structure of CalG2 (PDB code: 3RSC; protein shown in deep teal) is superimposed on the structure models of *Bc*GT1. (C, D) Close-up structures showing the *Bc*GT1 active site with UDP (C, D) and β-cellobiosyl 15HCM (C)- or β-gentiobiosyl 15HCM (D)-bound. The putative catalytic dyad of His14 and Asp106 can facilitate protonation of the glycosidic oxygen via one of two water molecules (red spheres). Water molecules were from the crystal structure of CalG2 (PDB code: 3RSC).

The atomic structure of *Bc*GT1 is not known, but a few tempered conclusions can be made based on the results of homology modeling (Figure 9), using the crystal structure of calicheamicin glycosyltransferase^47^ (CalG2, PDB code: 3RSC, sequence identity with *Bc*GT1, 28.5%) as the template. Based on sequence similarity according to the CAZy database (http://www.cazy.org/),^48^*Bc*GT1 is classified into glycosyltransferase family GT1. This is a large enzyme family involving natural product glycosyltransferases and Supporting Figure S14 shows a structure-based sequence alignment of *Bc*GT1 with several of such glycosyltransferases. The model suggests *Bc*GT1 to adopt the GT-B fold^47,49,50^ consisting of two β/α/β Rossman fold-like domains (see Figure 9A,B). The C-terminal domain, which binds the sugar nucleotide, is structurally well conserved and rigid. The N-terminal domain for acceptor substrate binding is more variable in sequence and structurally flexible. Dockings of UDP (Figure 9A) and UDP-glucose (Supporting Figure S15) indicate a well-defined stable binding of the uridine moiety in the C-terminal domain. The pyrophosphate moiety is bound more flexibly (Supporting Figure S15). The UDP docking is consistent with the experimental binding of thymidine-5′-diphosphate in CalG2. The glucosyl residue of UDP-glucose is positioned in the interdomain cleft (Supporting Figure S15). Docking models of the disaccharide-modified 15HCM products bound to the *Bc*GT1-UDP complex are shown in Figure 9C (cellobiosyl) and 9D (gentiobiosyl). The 15HCM β-d-glucoside part of the products is accommodated in the N-terminal domain. The terminal glucosyl residue protrudes into the interdomain cleft and its position in both products overlaps well with that of the glucosyl residue of UDP-glucose (Supporting Figure S16A,B). The ternary *Bc*GT1 complexes with UDP and disaccharide-modified product are plausible regarding catalysis. The catalytic dyad of His14 and Asp106 is positioned to provide protonic assistance, via intermediate water molecules, to the reversible cleavage of the β1,4- and β1,6-glycosidic bonds in the cellobiose- and gentiobiose-modified 15HCM (Figure 9C,D). While the His-Asp dyad is widely conserved among family GT1 glycosyltransferase,^47,49,50^ the intermediary waters are an interesting feature, as previously noted for CalG2.^47^ It is tempting to speculate that the relatively high hydrolase activity of *Bc*GT1 toward UDP-glucose may be connected to these water molecules. Considering the flexibility of the bound pyrophosphate group (Supporting Figure S15) mentioned above, the β-phosphate group of UDP is positioned reasonably (∼5.6 Å; Supporting Figure S16C,D) for attack on the anomeric carbon to form the α-configured UDP-glucose in a single displacement-like reaction.

The docking results underpin the remarkable permissiveness of *Bc*GT1 in the reaction with acceptor substrates. The enzyme binding pocket allows for the 15HCM portion of the disaccharide-modified products to be accommodated in two completely different orientations. The subterminal β-glucoside is thus positioned for glycosylation at O4 or O6 (Figure 9C,D). Supported by the literature on related natural product glycosyltransferases,^47,49,50^ the structure modeling of *Bc*GT1 suggests that a large and easily accessible binding pocket is crucial for permissive reactivity with acceptors representing a broad variety of chemical structures. It is also the basis for iterative glycosylation, requiring reaction with a nonsugar acceptor in the first step and with a sugar in the second. Like *Bc*GT1, glycosyltransferases performing multiple or iterative glycosylation reactions on acceptor substrates feature wide open and highly flexible binding pockets. Important examples are calicheamicin glycosyltransferases from *Micromonospora echinospora,*(47) the ginsenoside protopanaxadiol glycosyltransferase Bs-YjiC from *Bacillus subtilis,*(49) and the macrolide glycosyltransferase OleD from *Streptomyces antibioticus.*(50) Glycosyltransferases can bind acceptor substrates in different orientations for glycosylation.^51^

In conclusion, permissive glycosylation from UDP-glucose catalyzed by *Bc*GT1 is identified here for controlled β-glucosyl disaccharide modification of xenobiotic aglycones. A convenient synthetic route towards an important structural class of glycosylated products of detoxification metabolism is thus presented. The enzymatic conversion is performed in one pot without the requirement for isolation of intermediary products. It gives the desired disaccharide-modified product in high yield based on the aglycone provided. Two elements of the biotransformation are crucial for synthetic efficiency and practical utility: (1) *Bc*GT1 combines high β-selectivity in the glycosylation performed with broad specificity for the acceptor substrate used. Enzyme regioselectivity in the second step (β-glucoside glycosylation) of the iterative glycosylation sequence is relaxed, with β1,4 and β1,6-glycosylation occurring at a similar (2:1) frequency. However, the installment of both cellobiose and gentiobiose residues is of considerable synthetic interest, even in mixture, for the biological relevance that exactly these modifications have in the detoxification metabolism of xenobiotics.^18−21^ (2) Reaction engineering, to couple the *Bc*GT1 reaction with the reaction of sucrose synthase, is used to achieve two effects. First, UDP-glucose is supplied *in situ* at a suitable steady-state concentration that is constant during the whole conversion. Second, the UDP accumulating in the reaction is kept low. With sucrose used in excess, the mass action ratio from [UDP-glucose]/[UDP] is maintained to drive the iterative glycosylation from UDP-glucose to complete. Programmable synthesis of the disaccharide-modified product is thus enabled. The direct addition of UDP-glucose in excess cannot achieve the same: complex mixtures of monosaccharide and disaccharide-modified aglycones are obtained in a composition variable over time. The evidence from the current study can be broadly relevant in extending the synthetic scope of sugar nucleotide-dependent glycosyltransferases for controlled disaccharide modification. The strategy can be used flexibly (i.e., is not limited to UDP-glucose) by integrating sugar nucleotide-modifying enzymes (e.g., epimerases^36,42^) into the enzymatic cascade. Finally, the results presented inform glycosylation cascade reactions that involve glycosyltransferases operating in the reverse direction.^10,43^ The importance of controlling the mass action ratio of sugar nucleotide/nucleotide during the conversion is emphasized.

## Supporting Information Available

Optimized gene sequence of *Bc*GT1, NMR data of 15HCM β-d-glucosyl-β-d-glucosides (Tables S1 and S2); HPLC traces of 15HCM β-d-glucosyl-β-d-glucosides (Figure S1); mass analysis of 15HCM β-d-glucosyl-β-d-glucosides (Figures S2 and S3); NMR spectra of 15HCM β-d-glucosyl-β-d-glucosides (Figures S4–S9); reaction of 4MU with UDP-glucose (Figure S10); de-glycosylation of 4MU-β-d-glucoside after treatment of phosphatase (Figure S11); de-glycosylation of 15HCM-β-d-glucoside by *Bc*GT1 with UDP (Figure S12); hydrolysis of UDP-glucose by *Bc*GT1 (Figure S13); multiple sequence alignment of *Bc*GT1 and other glycosyltransferases of family GT1 (Figure S14); structural model of *Bc*GT1 bound with UDP-glucose (Figure S15); and structural interpretation of reverse glycosylation by *Bc*GT1 (Figure S16) (PDF)

## Abbreviations Used

*Bc*GT1: glycosyltransferase from *Bacillus cereus*
UDP: uridine 5′-diphosphate
15HCM: 15-hydroxy cinmethylin
NMR: nuclear magnetic resonance
MS: mass spectrometry
CM: cinmethylin
4MU: 4-methylumbelliferone
4NP: 4-nitrophenol
LB: Luria-Bertani
IPTG: isopropyl β-d-1-thiogalactopyranoside
DEAE FF: diethylaminoethanol-sepharose fast flow
*Gm*Susy: sucrose synthase from soybean *Glycine max*
SDS PAGE: sodium dodecyl sulfate polyacrylamide gel electrophoresis
HEPES: 4-(2-hydroxyethyl)-1-piperazineethanesulfonic acid)
DMSO: dimethyl sulfoxide
HPLC: high-performance liquid chromatography
UV–vis: ultraviolet–visible
HSQC: heteronuclear single quantum coherence
DEPT: distortionless enhancement by polarization transfer
COSY: correlation spectroscopy
HMBC: heteronuclear multiple bond correlation
ESI-MS: electrospray ionization mass spectrometry
TCEP: tris(2-carboxyethyl)phosphine
