# Supplementary material for: Controllable Iterative β-Glucosylation from UDP-Glucose by Bacillus cereus Glycosyltransferase GT1: Application for the Synthesis of Disaccharide-Modified Xenobiotics
Source: J Agric Food Chem. 2021 Nov 24;69(48):14630–42. doi: 10.1021/acs.jafc.1c05788 (PMC8662728; doi:10.1021/acs.jafc.1c05788)
Supplement: Supplementary file 1 — jf1c05788_si_001.pdf [file jf1c05788_si_001.pdf]

Controllable Iterative β-Glucosylation from UDP-Glucose by *Bacillus cereus* Glycosyltransferase GT1: Application to the Synthesis of Disaccharide-Modified Xenobiotics

Jihye Jung[a,b], Doreen Schachtschabel[c], Michael Speitling[c], Bernd Nidetzky[a,b,*]

[a] Austrian Centre of Industrial Biotechnology, A-8010 Graz, Austria

[b] Institute of Biotechnology and Biochemical Engineering, Graz University of Technology, NAWI Graz, A-8010 Graz, Austria

[c] BASF SE, Carl-Bosch-Strasse 38, 67056 Ludwigschafen, Germany

[*] Corresponding author. phone: +43-316-873-8400; e-mail: bernd.nidetzky@tugraz.at

Supporting Information

Table of contents

Optimized Gene Sequence of *Bc*GT1 used in this study

Supporting Table S1. NMR data of 15HCM β-D-glucosyl-(1→4)-β-D-glucoside.

Supporting Table S2. NMR data of 15HCM β-D-glucosyl-(1→6)-β-D-glucoside.

Supporting Figure S1. HPLC traces of 15HCM β-D-glucosyl-β-D-glucosides by using gradient and isocratic methods.

Supporting Figure S2. Mass data of 15HCM disaccharide product **P1**.

Supporting Figure S3. Mass data of 15HCM disaccharide product **P2**.

Supporting Figure S4. Chemical structure and ^{1}H-NMR spectra of disaccharide product **P1** (15HCM β-D-glucosyl-(1→4)-β-D-glucoside).

Supporting Figure S5. ^{13}C-NMR spectra of disaccharide product **P1** (15HCM β-D-glucosyl-(1→4)-β-D-glucoside).

Supporting Figure S6. HSQC-NMR spectrum of disaccharide product **P1** (15HCM β-D-glucosyl-(1→4)-β-D-glucoside).

Supporting Figure S7. H, H-COSY spectrum of disaccharide product **P1** (15HCM β-D-glucosyl-(1→4)-β-D-glucoside).

Supporting Figure S8. HMBC-spectrum of disaccharide product **P1** (15HCM β-D-glucosyl-(1→4)-β-D-glucoside).

Supporting Figure S9. NMR spectra of disaccharide product **P2** (15HCM β-D-glucosyl-(1→6)-β-D-glucoside).

Supporting Figure S10. Reaction of 4MU with *Bc*GT1 and UDP-glucose.

Supporting Figure S11. Phosphatase effect on de-glycosylation of 4MU-β-D-glucoside by *Bc*GT1 and internal nucleotide diphosphate.

Supporting Figure S12. Reverse glycosylation of 15HCM-β-D-glucoside by *Bc*GT1 with UDP.

Supporting Figure S13. Hydrolysis of UDP-glucose by *Bc*GT1.

Supporting Figure S14. Structure-based sequence alignment of *Bc*GT1 with other family GT1 glycosyltransferases.

Supporting Figure S15. Structural model of *Bc*GT1 with UDP-glucose.

Supporting Figure S16. Structural interpretation of reverse glycosylation by *Bc*GT1.

Reference

Optimized Gene Sequence of *Bc*GT1 used in this study

ATGGCCAATGTTCTGGTTATTAATTTCCCTGGTGAAGGTCATATTAATCCGACCCTGGCAATT
GTTAGTGAACTGATTCGCCGTGGCGAAACCGTGGTGAGTTATTGTATTGAAGATTATCGTAA
GAAGATCGAAGCAACCGGCGCACAGTTTCGCGTGTTTGAAAATTTTCTGAGTCAGATTAAC
ATCATGGAACGTGTTAATGAAGGTGGTAGTCCGCTGACCATGCTGAGCCACATGATGGAAG
CAAGTGAACGCATTGTGACCCAGATTGTTGAAGAAACCAAAGGCGAAAAATATGATTATCT
GATCTATGATAACCACTTCCCGGTTGGTCGCATTATTGCAAATGTGCTGAAACTGCCGAGCG
TGAGTAGCTGTACCACCTTTGCATTCAATCAGTATATTACCTTTAACGATGAGCATGAAAGC
CGTGAAGTTGATGAAACCAATCCGCTGTATCAGAGTTGTCTGGCCGGCATGGAAAAATGG
AATAAGCAGTATGGTATGAAATGCAATAGTATGTATGACATTATGAACCATCCGGGTGACAT
TACCATTGTTTATACCAGTAAAGAATACCAGCCGCGCAGCGATGTGTTTGATGAAAGTTATA
AATTCGTGGGTCCGAGTATTGCCACCCGCAAAGAAGTGGGCAGCTTTCCGATGGAAGATCT
GAAAGATGAAAAACTGATTTTCATCAGTATGGGTACCGTGTTTAATGAACAGCCGGAACTG
TATGAAAAATGCTTTGAAGCATTCAAAGACGTTGAAGCAACCGTGGTTCTGGTTGTGGGTA
AAAAGATTAATATTAGTCAGTTCGAGAACATCCCGAATAATTTTAAACTGTATAACTACGTG
CCGCAGCTGGAACTGCTGCAGTATGCCGATGTGTTTGTTACCCACGGTGGTATGAATAGTA
GCAGCGAAGCCCTGTATTATGGCGTGCCGCTGGTTGTGATTCCGGTTACCGGCGATCAGCC
GCTGGTTGCCAAACGTGTGAATGAAGTTGGCGCCGGCATTCGCCTGAATCGTAAAGAACT
GACCAGCGAAATGCTGCGCGAAAGTGTGAAAAAAGTGATGGATGATGTTACCTTTAAGGA
AAAAAGTCGTAAAGTTGGCGAAAGTCTGCGCAATGCAGGTGGCTATAATCGTGCCGTTGAT
GAAATTCTGAAAATGAATAGTTACAGCAAGCTGAAATGA

Supporting Table S1. NMR data of 15HCM β-D-glucosyl-(1→4)-β-D-glucoside. Data were obtained from Supporting Figure S4 – S8.

Atom#	XHn	C Shift	H Shift	H Multiplicity	COSY	H to C HMBC	C to H HMBC
**1**	CH_2	33.57	1.4	m	1	17, 5	4
**1**	CH_2	33.57	1.47	m	1	17, 5	
**2**	CH_2	31.62	1.42	m		18	
**3**	C	87.65				19, 20, 4, 4, 5	
**4**	CH_2	41.63	1.42	m	4	1, 18	1, 3
**4**	CH_2	41.63	1.96	m	4, 5	1, 18	18, 6, 3
**5**	CH	82.88	3.6	m	4	8, 8	1, 8, 6, 3
**6**	C	84.82				17, 4, 5	
**8**	CH_2	67.02	4.55	d (12.6)	8	5, 14	5, 14, 9
**8**	CH_2	67.02	4.42	d (12.6)	8	5, 14	5, 14, 10, 9
**9**	C	137.05				8, 8, 15, 15, 13, 11	
**10**	C	135.62 [a]				8, 15, 15, 12, 14	
**11**	CH	128.37	7.46	br d (7.2)	12	15, 15, 13, 13	15, 13, 9
**12**	CH	127.16	7.27	m	13, 11	14, 13	13, 10
**13**	CH	127.47	7.29	m	12, 14	12, 11	11, 9
**14**	CH	127.85	7.37	br d (7.2)	13	8, 8, 12	8, 12, 10
**15**	CH_2	67.45	4.62	m	15	11	22, 11, 10, 9
**15**	CH_2	67.45	4.86	m	15	11	22, 11, 10, 9
**17**	CH_3	16.58	1.34	2 s			1, 6
**18**	CH	32.18	1.96	m	19, 20	19, 20, 2	19, 20, 2, 4
**19, 20**	CH_3	18.04	0.9	t (6.59, 6.59)	18	18	18, 3
**22**	CH	101.88	4.25	m	27	27, 15, 15	
**24**	CH	74.93	3.27	m	25, 26, 29	27, 25, 26, 29, 29	
**25**	CH	80.57	3.34 [a]	m	24, 29	26, 29, 33	29, 24, 33
**26**	CH	75.04	3.32 [a]	m	27, 24	27, 25, 29	27, 25
**27**	CH	73.21	3.09	br t (8.06, 8.06)	25, 26, 22		24, 22
**29**	CH_2	60.46	3.77	m	29	25, 26	24, 25, 26
**29**	CH_2	60.46	3.65	m	24, 29	25, 26	24
**33**	CH	103.22	4.26	m	38	38, 25, 26	37, 25, 26
**35**	CH	76.81	3.19	m	36, 40	36, 40	40
**36**	CH	70.03	3.05	m	37, 35	37, 40	40, 35
**37**	CH	76.45	3.16	m	38, 36	38, 33	36, 38
**38**	CH	73.3	2.99	t (8.41, 8.41)	37, 33	37	37, 33
**40**	CH_2	61.01	3.4	m	35, 40	36, 35	35
**40**	CH_2	61.01	3.69	m	40	36, 35	36

[a] The data were obtained from 2D-NMR spectra (Supporting Figure S6-8).

Supporting Table S2. NMR data of 15HCM β-D-glucosyl-(1→6)-β-D-glucoside.[a]

Atom#	XHn	C Shift	H Shift	H to C HMBC	C to H HMBC
**1**	CH_2	33.78	1.4	17, 2, 4	2, 5, 3
**1**	CH_2	33.78	1.48	17, 2, 4	2, 5
**2**	CH_2	31.83	1.42	1, 2, 4	5, 3
**3**	C	88.06		19, 20, 2, 4, 5	
**4**	CH_2	41.99	1.42	18	2, 5, 3
**4**	CH_2	41.99	1.96	18	19, 20, 6, 3
**5**	CH	83.25	3.61	8, 8	1, 8, 3
**6**	C	85.27		17	
**8**	CH_2	67.22	4.43	5, 14	5, 13, 14, 9
**8**	CH_2	67.22	4.48	5, 14	13, 14, 9
**9**	C	137.96		8, 15, 13, 11	
**10**	C	135.81		12, 14	
**11**	CH	129.21	7.45	13	15, 13, 14, 9
**12**	CH	127.45	7.26	14	13, 14, 10
**13**	CH	127.84	7.29	8, 11, 12	11, 9
**14**	CH	127.84	7.39	8, 11, 12	8, 12, 10
**15**	CH_2	67.42	4.58	22, 11	22, 11, 10, 9
**15**	CH_2	67.42	4.86	22, 11	22, 11, 10
**17**	CH_3	16.97	1.34		1, 6
**18**	CH	32.61	1.96	19, 20	19, 20, 4, 3
**19, 20**	CH_3	18.33	0.9	19, 20, 18	19, 20, 18, 3
**22**	CH	102.22	4.19	15, 15	15
**29**	CH_2	68.98	3.60	33	24, 33
**29**	CH_2	68.98	4.01	33	33
**33**	CH	103.79	4.30	29, 29	29

[a] The data were obtained from HSQC and HMBC spectra. Signals of C24 – C27, C35 – C38, and C40 were overlapped with signals from the sugars of trisaccharides. The primary glycosidic linkage with hydroxyl group of 15HCM (C22 and C15) and iterative β(1→6) glycosidic linkage (C33 and C29) are shown in Supporting Figure S9.

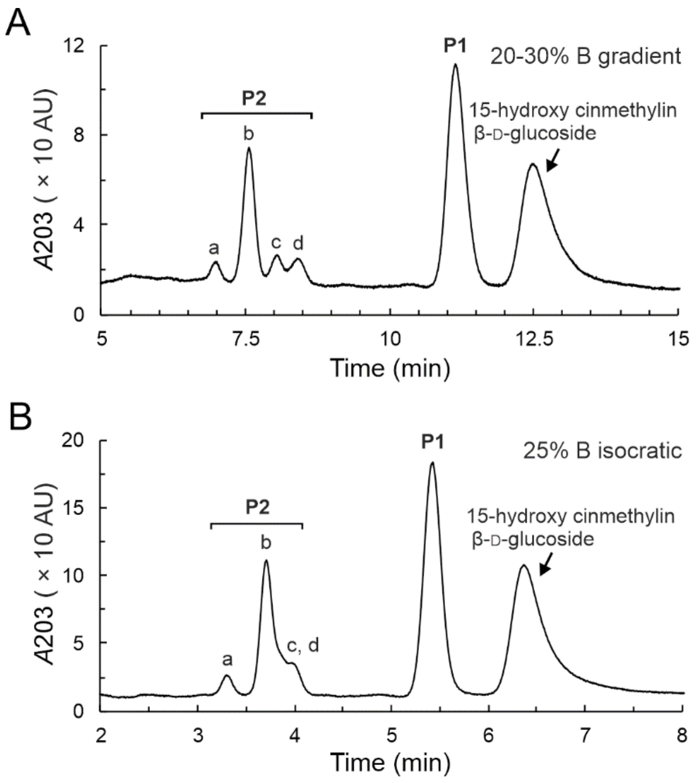

Supporting Figure S1. HPLC traces of 15HCM β-D-glucosyl-β-D-glucosides by using gradient and isocratic methods. Acetonitrile and water were used. Formic acid (0.1% v/v) was added into acetonitrile and water, respectively. Separation of product **P2** was improved neither with a gradient method of 20-30% acetonitrile in water for 40 min (upper), nor with an isocratic method of 25% acetonitrile for 10 min (bottom).

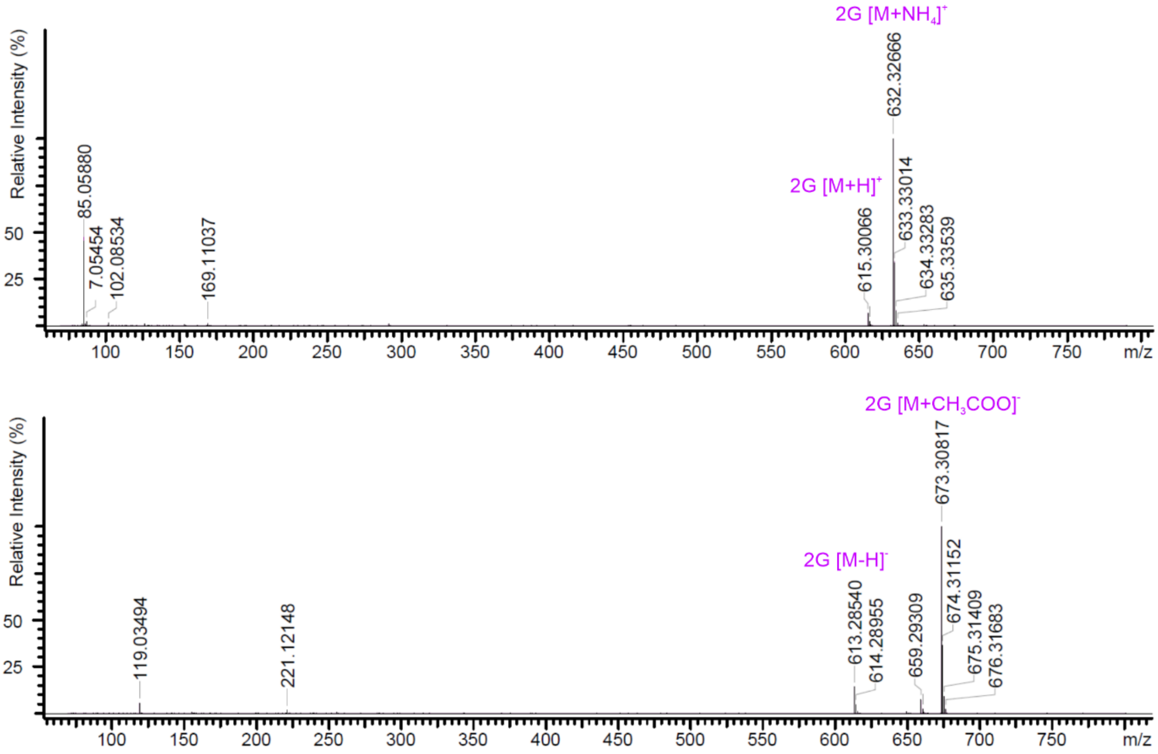

Supporting Figure S2. Mass data of 15HCM disaccharide product **P1**. Positive (upper) and negative (bottom) modes were used. Detected mass were consistent with 15HCM disaccharides (2G, mass 614; $[M+H]^+$, 615; $[M+NH_4]^+$, 632; $[M-H]^-$, 613; $[M+CH_3COO]^-$, 673).

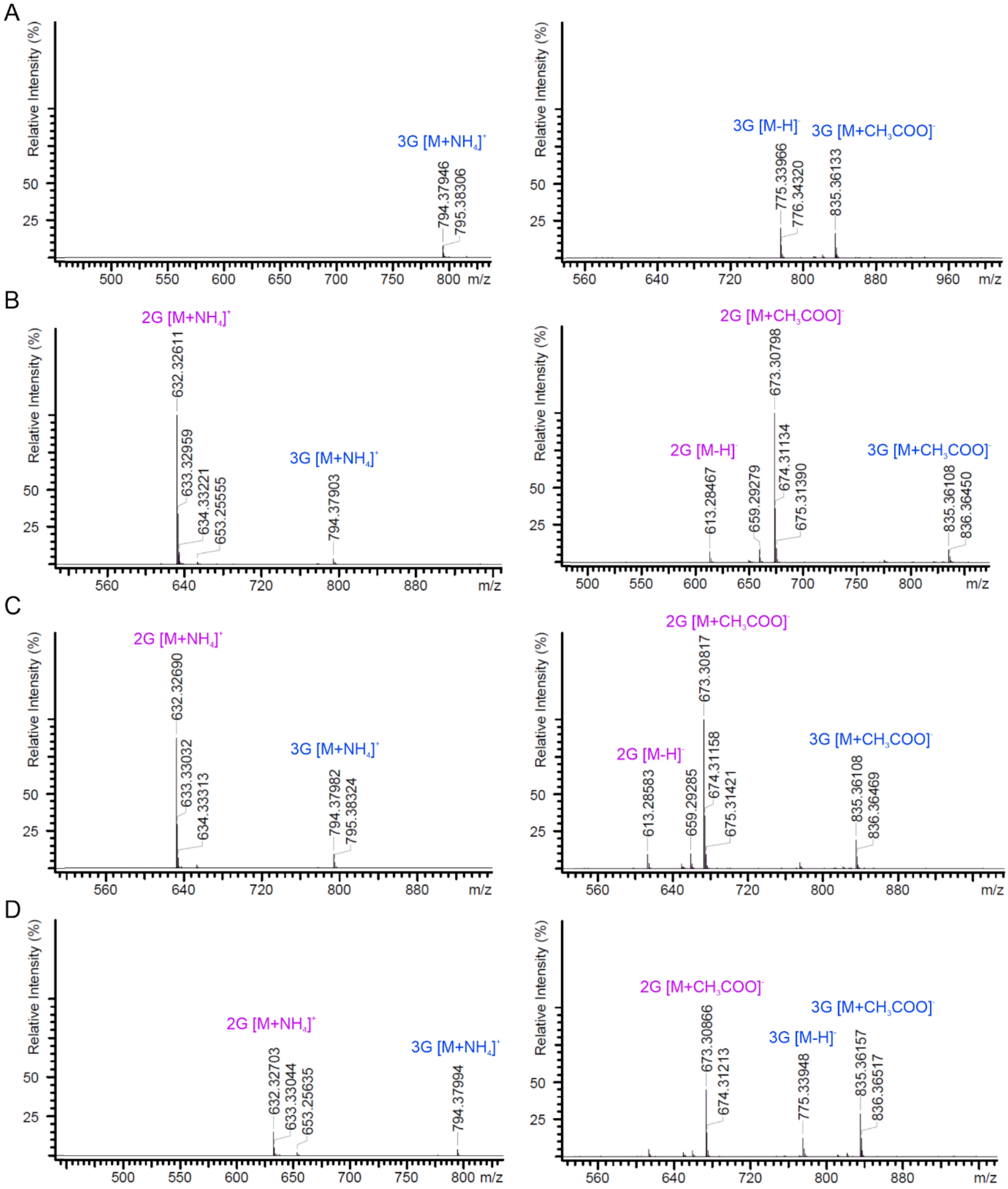

Supporting Figure S3. Mass data of 15HCM disaccharide product **P2**. Positive (left phanels) and negative (right panels) modes were used. Disaccharides (2G, mass 614; $[M+NH_4]^+$, 632; $[M-H]^-$, 613; $[M+CH_3COO]^-$, 673) and trisaccharides (3G, mass 776; $[M+NH_4]^+$, 794; $[M-H]^-$, 775; $[M+CH_3COO]^-$, 835) were detected. (A) **P2**-a. (B) **P2**-b. (C) **P2**-c. (D) **P2**-d.

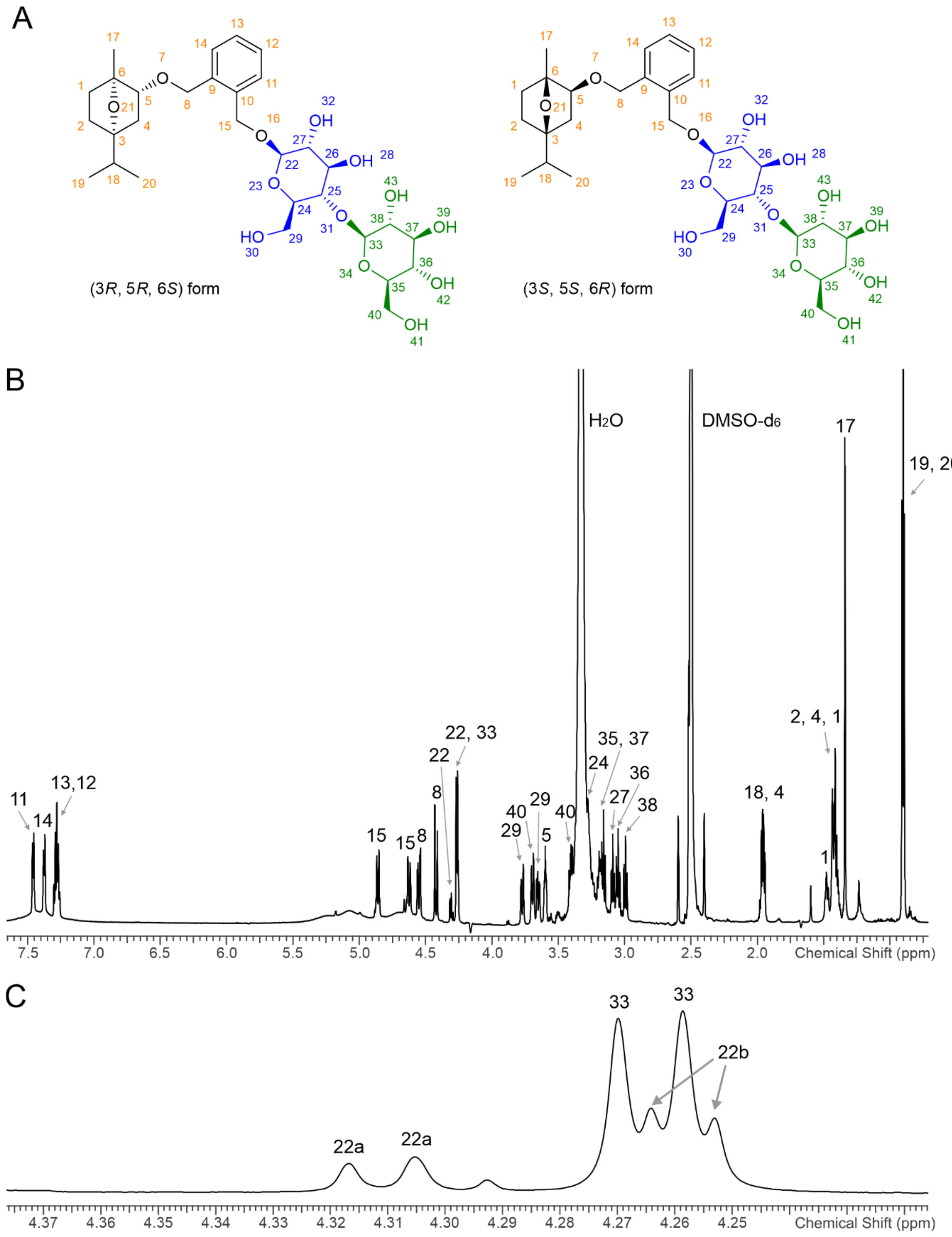

Supporting Figure S4. Chemical structure and ^{1}H-NMR spectra of disaccharide product **P1** (15HCM β-D-glucosyl-(1→4)-β-D-glucoside). DMSO-d_6 was used as solvent. (A) The diastereomeric structures of 15HCM β-D-glucosyl-(1→4)-β-D-glucoside with atom numbers. (B) ^{1}H-NMR. (C) Proton peaks of C22 and C33 of the diastereomeric 15HCM β-D-glucosyl-(1→4)-β-D-glucoside in ^{1}H-NMR. The proton peaks of C22 of the diastereomeric structures (22a and 22b) were shown at a ratio of approximately 1:1. The proton peaks of C33 of the diastereomeric structures were hardly observed, plausibly because of their considerable distance from the chiral center C5.

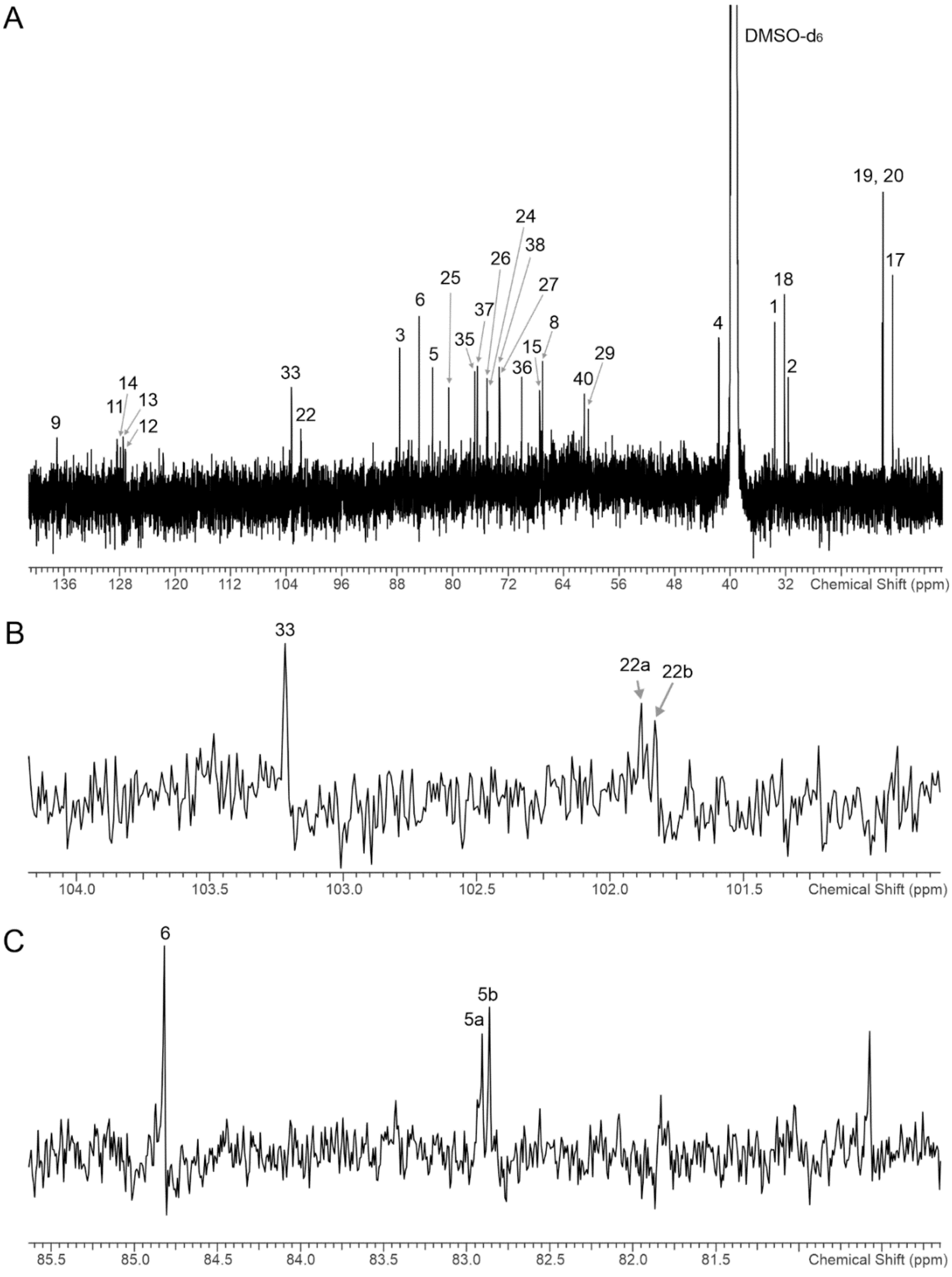

Supporting Figure S5. ^{13}C-NMR spectra of disaccharide product **P1** (15HCM β-D-glucosyl-(1→4)-β-D-glucoside). DMSO-d$_6$ was used as solvent. (A) ^{13}C-NMR. (B) Carbon peaks of C22 and C33 of the diastereomeric 15HCM β-D-glucosyl-(1→4)-β-D-glucoside in ^{13}C-NMR. The carbon peaks of C22 (22a and 22b) of the diastereomeric structures are observed. The carbon peaks of C33 of the diastereomeric structures were hardly observed, because of their considerable distance from the chiral center C5. (C) Carbon peaks of C5 of the diastereomeric 15HCM β-D-glucosyl-(1→4)-β-D-glucoside in 13C-NMR. The carbon peaks of C5 of the diastereomeric structures (5a and 5b) are shown.

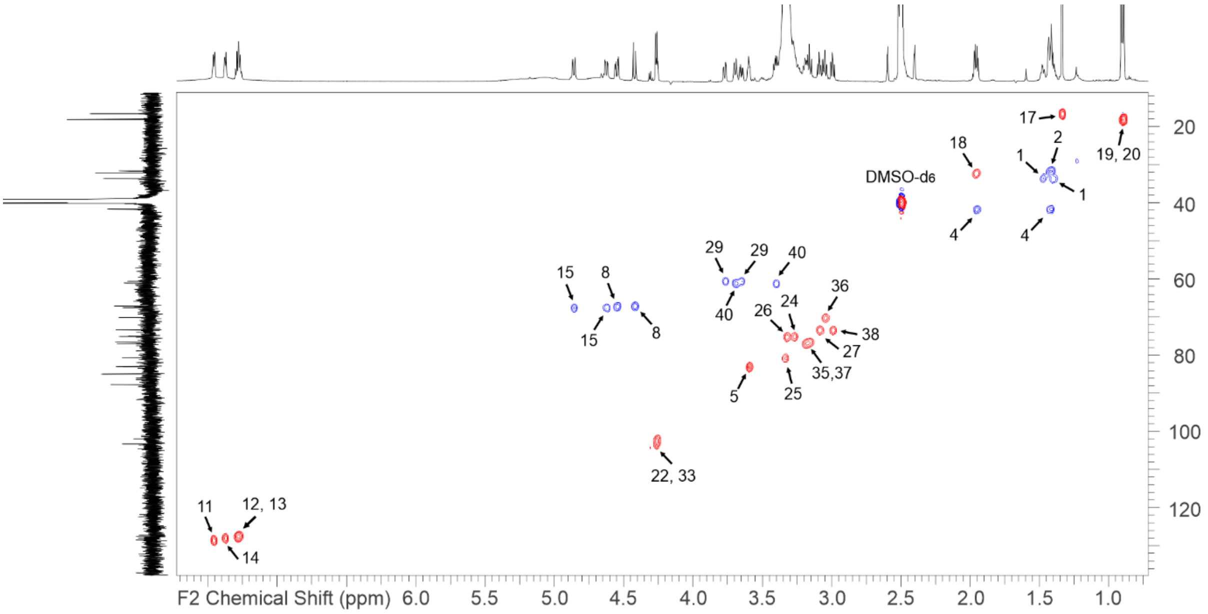

Supporting Figure S6. HSQC-NMR spectrum of disaccharide product **P1** (15HCM β-D-glucosyl-(1→4)-β-D-glucoside). DMSO-d_6 was used as solvent.

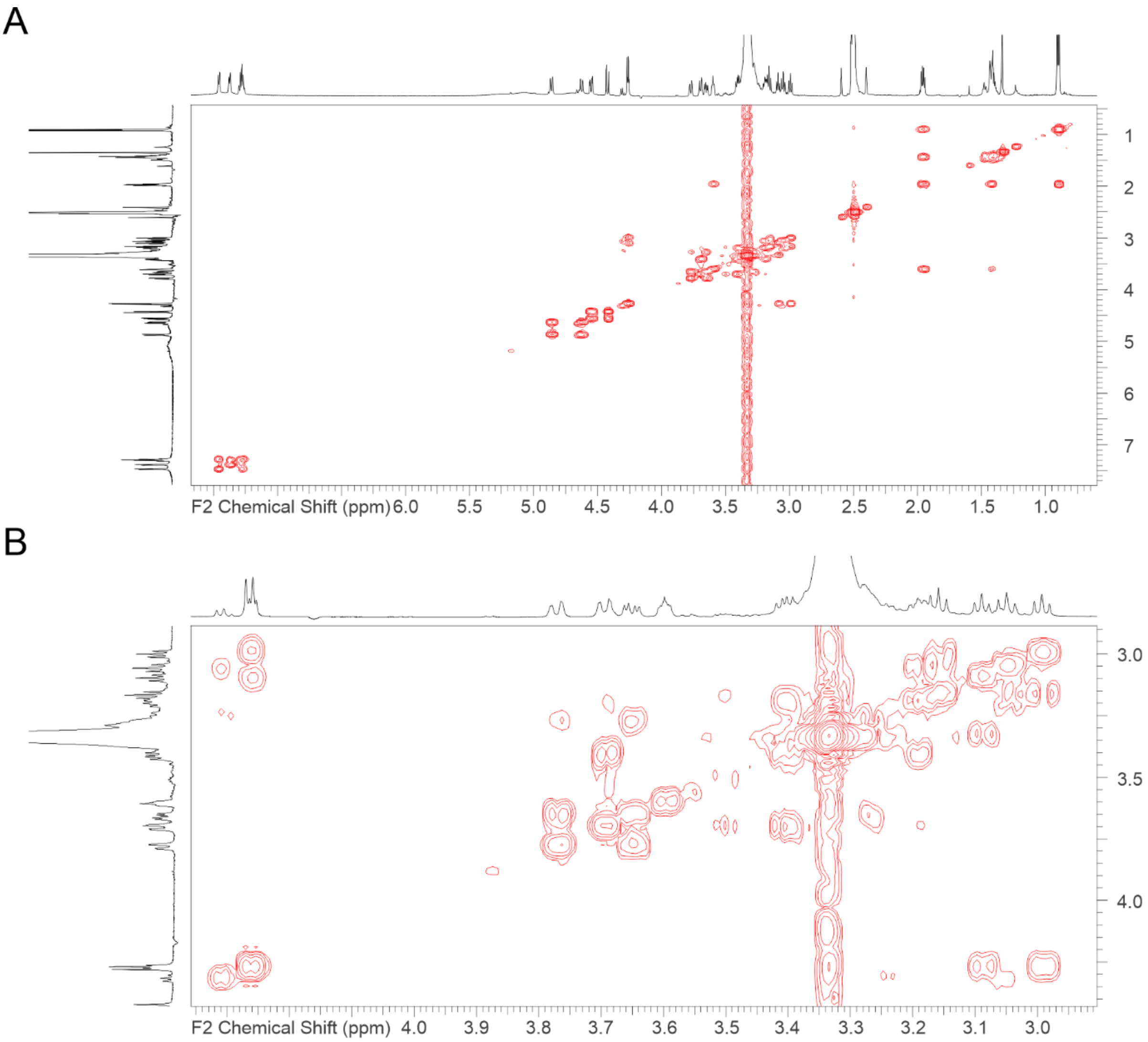

Supporting Figure S7. H, H-COSY spectrum of disaccharide product **P1** (15HCM β-D-glucosyl-(1→4)-β-D-glucoside). DMSO-d_6 was used as solvent. (A) Full spectrum. (B) Close-up of proton peaks of sugars.

A

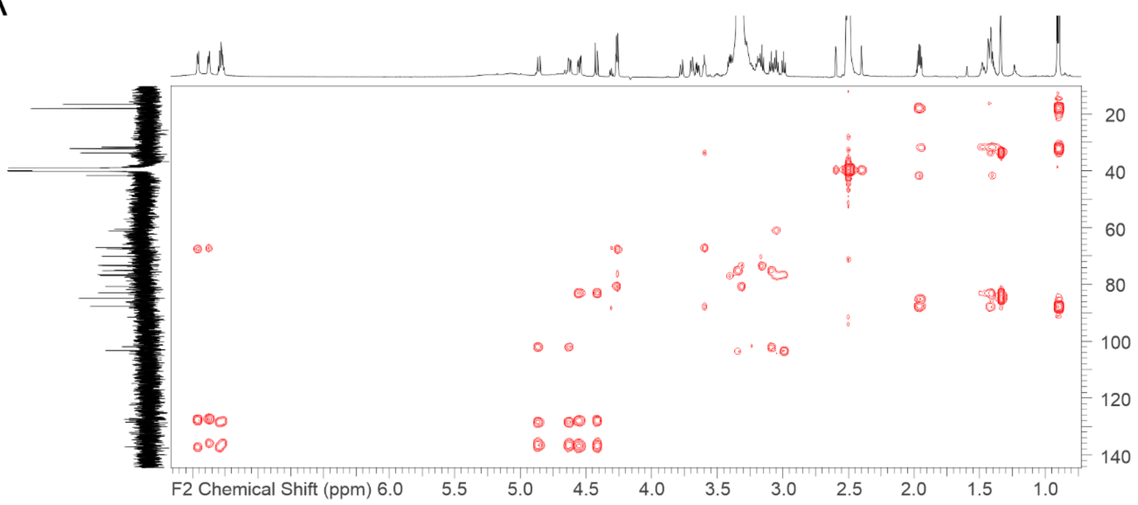

B

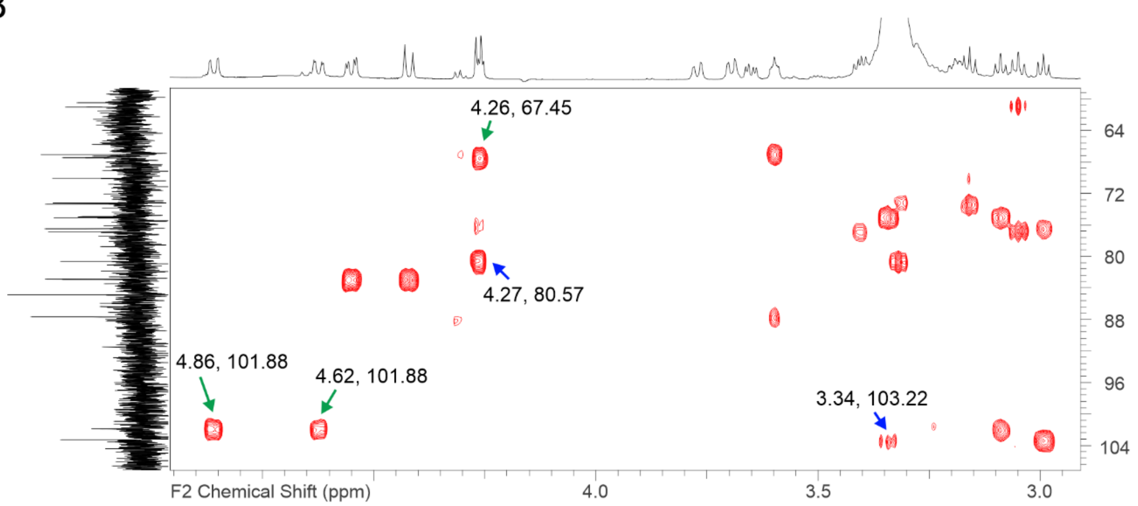

Supporting Figure S8. HMBC-spectrum of disaccharide product **P1** (15HCM β-D-glucosyl-(1→4)-β-D-glucoside). DMSO-d_6 was used as solvent. (A) Full spectrum. (B) Glycosidic linkages of 15HCM β-D-glucosyl-(1→4)-β-D-glucoside. Primary glycosidic linkage with hydroxyl group of 15HCM (green arrows, C15 and C22) and iterative β(1→4) glycosidic linkage (blue arrows, C25 and C33) are shown. 1H and 13C chemical shifts (ppm) are shown.

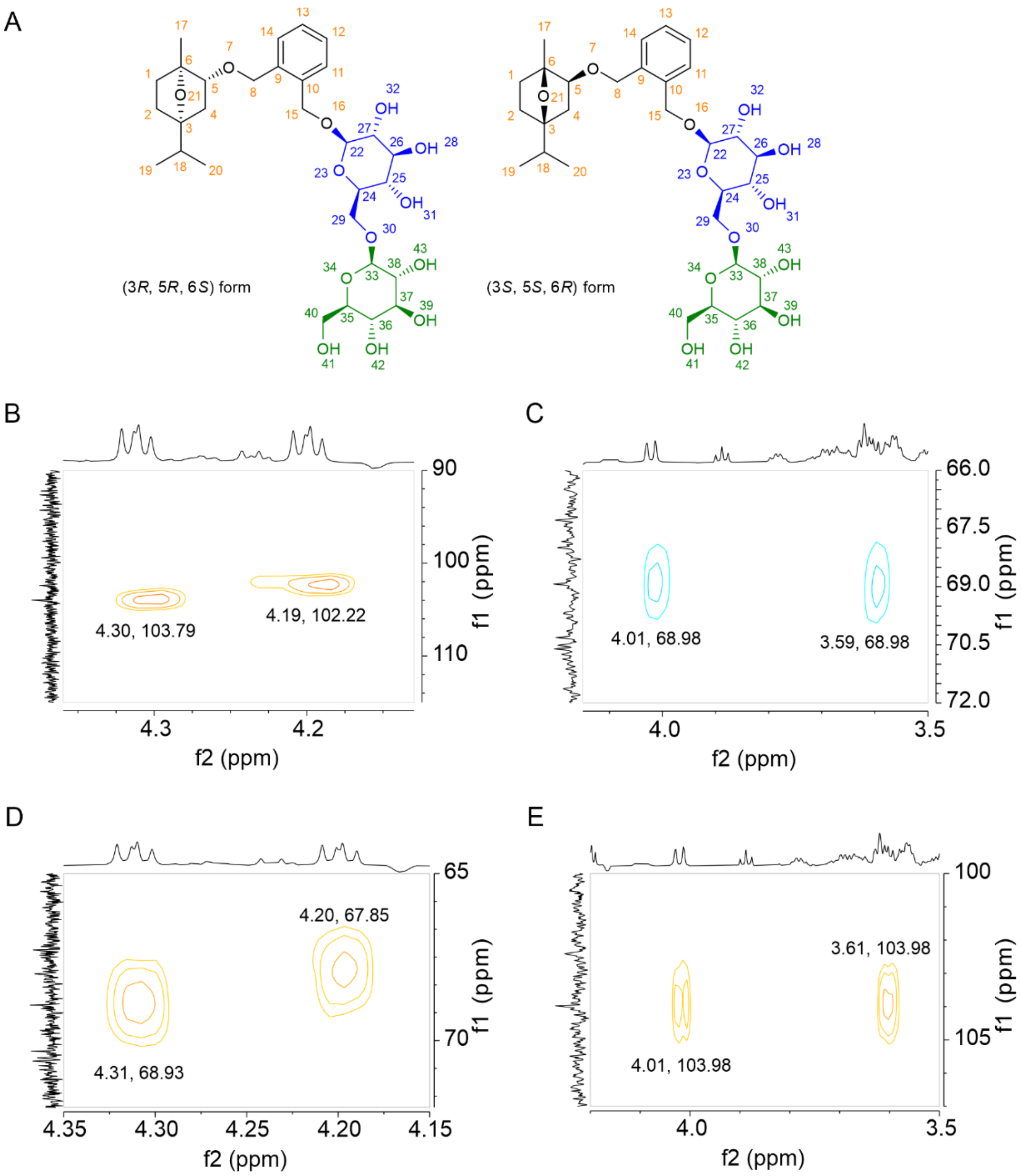

Supporting Figure S9. NMR spectra of disaccharide product **P2** (15HCM β-D-glucosyl-(1→6)-β-D-glucoside). DMSO-d_6 was used as solvent. Carbon spectra show lower signal intensity because product **P2** (~ 0.33 mg) was 2.2-fold lower amount than product **P1** (~ 0.73 mg). (A) The diastereomeric structures of 15HCM β-D-glucosyl-(1→6)-β-D-glucoside with atom numbers. (B-C) Relevant signals from C22, C29, and C33 of 15HCM β-D-glucosyl-(1→6)-β-D-glucoside in HSQC spectra. Signals from C22 (B, CH, 4.19 ppm, 102.22 ppm), C29 (C, CH_2, 3.60 ppm/4.01 ppm, 68.98 ppm), and C33 (B, CH, 4.30 ppm, 103.79 ppm) are shown. The proton peaks of C22 and C33 of the diastereomeric structures are shown. (D-E) Glycosidic linkages of 15HCM β-D-glucosyl-(1→6)-β-D-glucoside in HMBC spectra. The primary glycosidic linkage with hydroxyl group of 15HCM (D, proton of C22, 4.20 ppm, carbon of C15, 67.85 ppm) and iterative β(1→6) glycosidic linkage (D, proton of C33, 4.31 ppm, carbon of C29, 68.93 ppm; E, proton of C29, 3.61 ppm and 4.01 ppm, carbon of C33, 103.98 ppm) are shown.

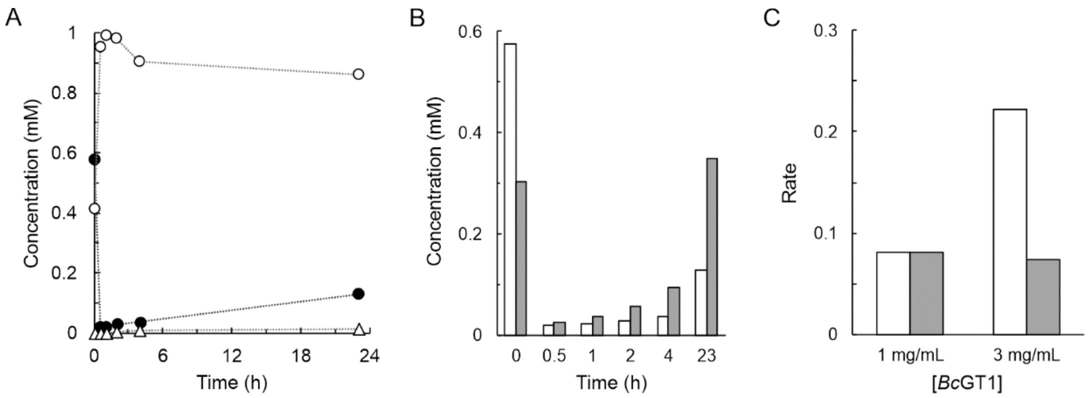

Supporting Figure S10. Reaction of 4MU with *Bc*GT1 and UDP-glucose. (A) Time-courses of the reaction with *Bc*GT1 (1 mg/mL). Closed circles are 4MU. Open circles and triangles are 4MU-β-D-glucosides and 4MU-disaccharides, respectively. (B) Concentration of 4MU incubated with *Bc*GT1 (white bar, 1 mg/mL; gray bar, 3 mg/mL) for 0 – 23 h. (C) Re-formation rates of 4MU from 4MU-β-D-glucoside by *Bc*GT1 (1 mg/mL, 3 mg/mL). Rates calculated from 4 h to 23 h are shown (white bar, mU/mL; gray bar, mU/mg).

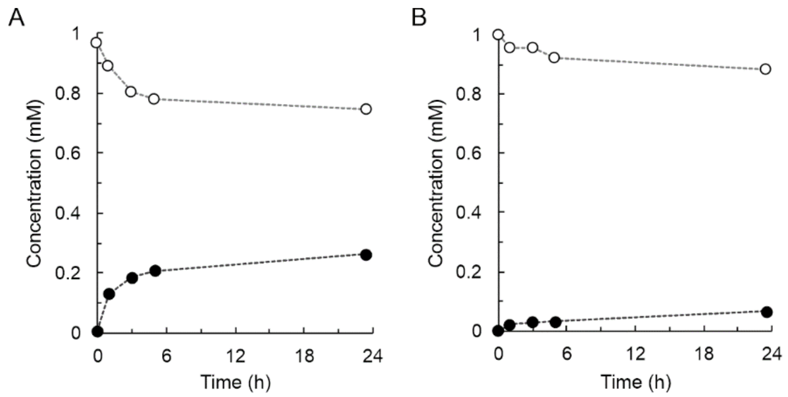

Supporting Figure S11. Phosphatase effect on de-glycosylation of 4MU-β-D-glucoside by *Bc*GT1 and internal nucleotide diphosphate. Open circles are 4MU-β-D-glucoside. Closed circles are 4MU. (A) Reaction without treatment of phosphatase. (B) Reaction after treatment of phosphatase.

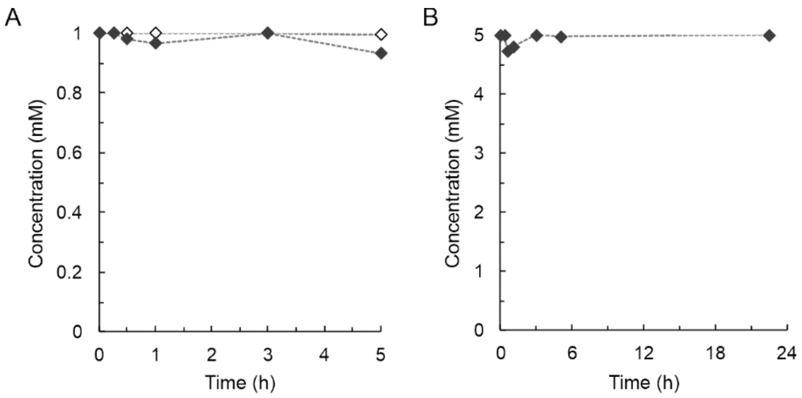

Supporting Figure S12. Reverse glycosylation of 15HCM-β-D-glucoside by *Bc*GT1 with UDP. (A) Reactions of 15HCM-β-D-glucoside (1 mM) and UDP (1 mM) in *Bc*GT1 (1 mg/mL) solution at pH 7.0 and pH 9.0. Sodium phosphate buffer (100 mM, pH 7.0; open diamond) and Tris buffer (50 mM, pH 9.0; closed diamond) were used. $MgCl_2$ (5 mM) was used. (B) Reactions of 15HCM-β-D-glucoside and *Bc*GT1 with UDP regeneration system. The corresponding reaction at pH 9.0 was conducted with UDP released from the reaction of *Gm*Susy with fructose and UDP-glucose.

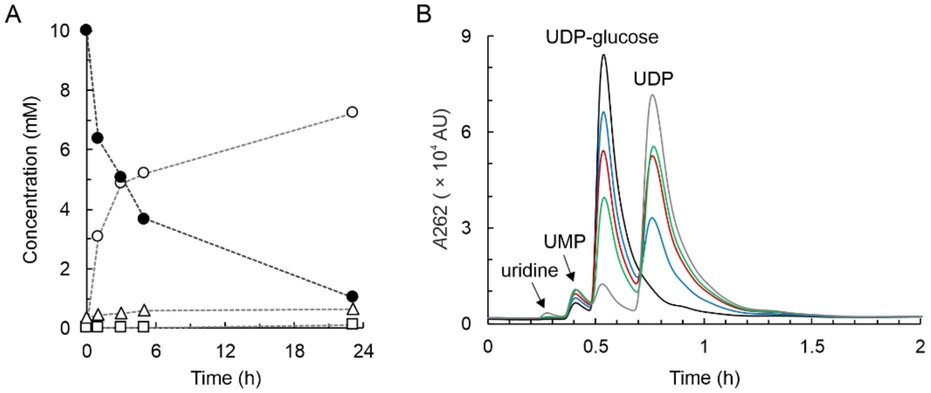

Supporting Figure S13. Hydrolysis of UDP-glucose by *Bc*GT1. The enzyme *Bc*GT1 (5 mg/mL) in sodium phosphate buffer (100 mM, pH 7.4) containing $MgCl_2$ (5 mM) was used. UDP-glucose was 10 mM. Temperature was 37 °C. (A) Time course of UDP-glucose hydrolysis. Closed circles are UDP-glucose. Open circles are UDP. Open triangles and squares are UMP and uridine, respectively. (B) HPLC traces of UDP-glucose hydrolysis (0 h, black; 1 h, blue; 3 h, red; 5 h, green; 23 h, gray).

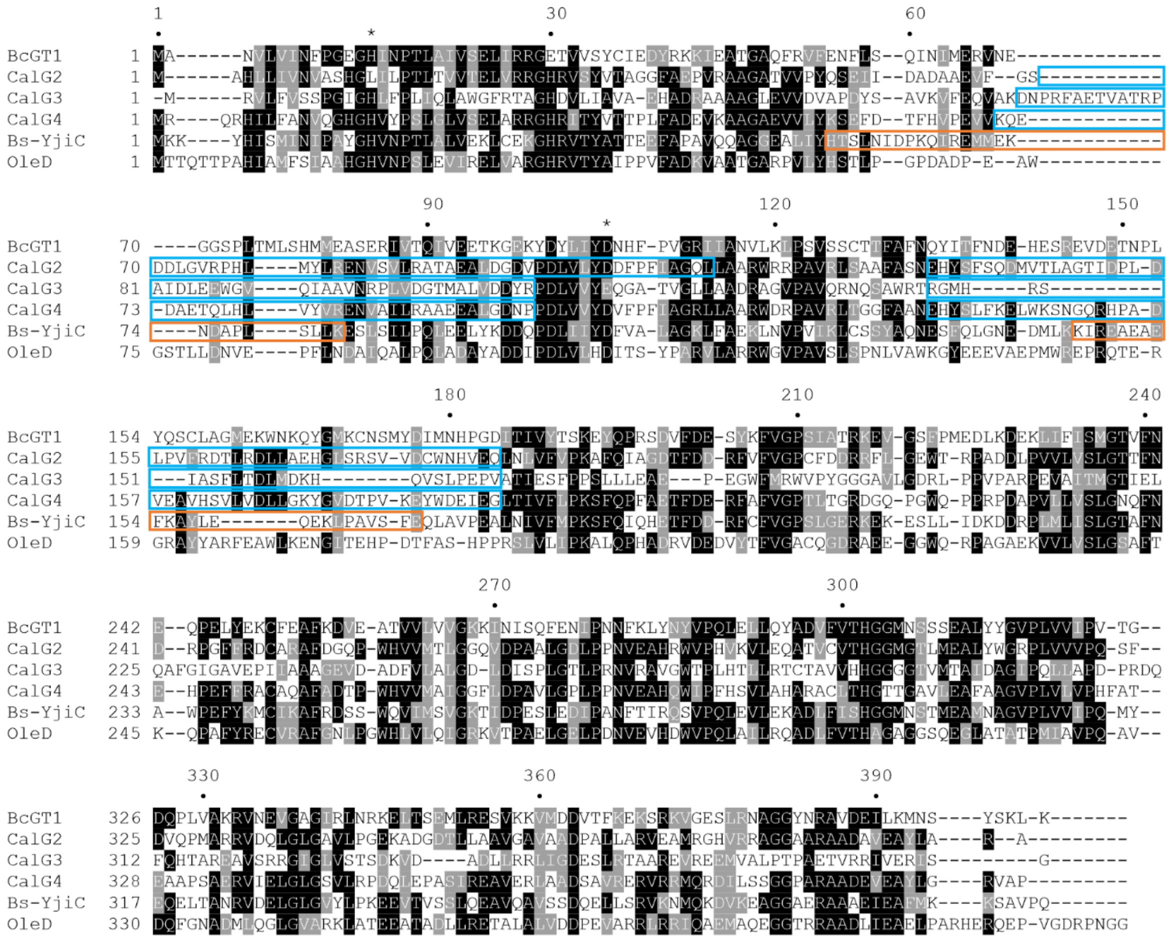

Supporting Figure S14. Structure-based sequence alignment of *Bc*GT1 with other family GT1 glycosyltransferases. Calicheamicin glycosyltransferases from *Micromonospora echinospora* (CalG2, PDB code: 3RSC; CalG3, PDB code: 3OTI; CalG4, PDB code: 3IA7)[1], glycosyltransferase from *Bacillus subtilis* (Bs-YjiC, PDB code: 7BOV and 6KQX)[2,3], and macrolide glycosyltransferase from *Streptomyces antibioticus* (OleD, PDB code: 2IYF)[4]. Structural alignment (expresso) in T-COFFEE was used[5,6]. Catalytic residues are marked with asterisks. Acceptor binding sites (showing structural variation in the calicheamicin glycosyltransferases) are marked with blue boxes. Sequence parts of Bs-YjiC not resolved in the crystal structure are marked with an orange box.

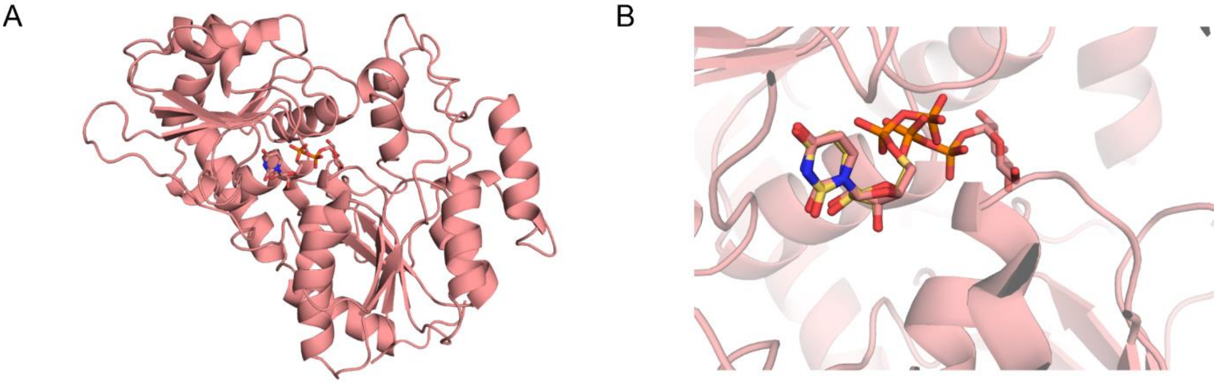

Supporting Figure S15. Structural model of *Bc*GT1 with UDP-glucose. (A) UDP-glucose bound form of *Bc*GT1 (light pink). (B) Close-up structure of sugar nucleotide binding site in *Bc*GT1. UDP-glucose (light pink) and UDP (yellow orange) are shown.

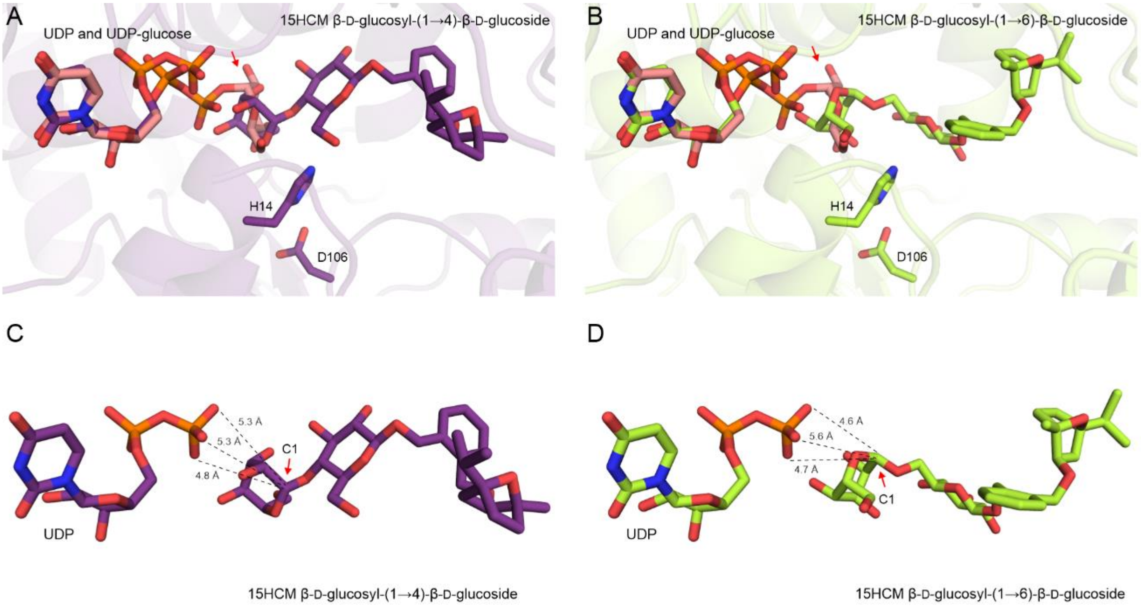

Supporting Figure S16. Structural interpretation of reverse glycosylation by *Bc*GT1. (A-B) Close-up structures of *Bc*GT1 active site with UDP-glucose (A, B; ligand shown in light pink) and β-cellobiosyl 15HCM (A, with UDP, protein and ligands shown in violet purple) or β-gentiobiosyl 15HCM (B, with UDP, protein and ligands shown in lemon). Red arrows indicate the superimposed glucosyl residues. (C-D) Potential attack of β-phosphate group of UDP on the anomeric carbon (red arrow, C1) of β-cellobiosyl 15HCM (C) or β-gentiobiosyl 15HCM (D).

Reference

(1) Chang, A.; Singh, S.; Helmich, K. E.; Goff, R. D.; Bingman, C. A.; Thorson, J. S.; Phillips, G. N. Complete set of glycosyltransferase structures in the calicheamicin biosynthetic pathway reveals the origin of regiospecificity. *Proc. Natl. Acad. Sci. U. S. A.* **2011**, *108*, 17649–17654.

(2) Dai, L.; Qin, L.; Hu, Y.; Huang, J.; Hu, Z.; Min, J.; Sun, Y.; Guo, R.-T. Structural dissection of unnatural ginsenoside-biosynthetic UDP-glycosyltransferase Bs-YjiC from *Bacillus subtilis* for substrate promiscuity. *Biochem. Biophys. Res. Commun.* **2021**, *534*, 73–78.

(3) Liu, B.; Zhao, C.; Xiang, Q.; Zhao, N.; Luo, Y.; Bao, R. Structural and biochemical studies of the glycosyltransferase Bs-YjiC from *Bacillus subtilis*. *Int. J. Biol. Macromol.* **2021**, *166*, 806–817.

(4) Bolam, D. N.; Roberts, S.; Proctor, M. R.; Turkenburg, J. P.; Dodson, E. J.; Martinez-Fleites, C.; Yang, M.; Davis, B. G.; Davies, G. J.; Gilbert, H. J. The crystal structure of two macrolide glycosyltransferases provides a blueprint for host cell antibiotic immunity. *Proc. Natl. Acad. Sci. U. S. A.* **2007**, *104*, 5336–5341.

(5) Di Tommaso, P.; Moretti, S.; Xenarios, I.; Orobitg, M.; Montanyola, A.; Chang, J.-M.; Taly, J.-F.; Notredame, C. T-Coffee: a web server for the multiple sequence alignment of protein and RNA sequences using structural information and homology extension. *Nucleic Acids Res.* **2011**, *39*, W13–W17.

(6) Armougom, F.; Moretti, S.; Poirot, O.; Audic, S.; Dumas, P.; Schaeli, B.; Keduas, V.; Notredame, C. Expresso: automatic incorporation of structural information in multiple sequence alignments using 3D-coffee. *Nucleic Acids Res.* **2006**, *34*, W604–W608.
